# *Acinetobacter baumannii* from grass: novel but non-resistant clones

**DOI:** 10.1099/mgen.0.001054

**Published:** 2023-07-13

**Authors:** Valeria Mateo-Estrada, Ciara Tyrrell, Benjamin A. Evans, Alejandro Aguilar-Vera, David Drissner, Santiago Castillo-Ramirez, Fiona Walsh

**Affiliations:** ^1^​ Programa de Genómica Evolutiva, Centro de Ciencias Genómicas, Universidad Nacional Autónoma de México, Cuernavaca, Mexico; ^2^​ Department of Biology, The Kathleen Lonsdale Human Health Institute, Maynooth University, Maynooth, Co. Kildare, Ireland; ^3^​ Norwich Medical School, University of East Anglia, Norwich, UK; ^4^​ Department of Life Sciences, Albstadt-Sigmaringen University, 72488 Sigmaringen, Germany

**Keywords:** Genomic epidemiology, *Acinetobacter baumannii*, antibiotic resistance, grass, international clones, One Health, non-clinical environments

## Abstract

*
Acinetobacter baumannii* is one the most worrisome nosocomial pathogens, which has long been considered almost mainly as a hospital-associated bacterium. There have been some studies about animal and environmental isolates over the last decade. However, little effort has been made to determine if this pathogen dwells in the grass. Here, we aim to determine the evolutionary relationships and antibiotic resistance of clones of *A. baumannii* sampled from grass to the major human international clones and animal clones. Two hundred and forty genomes were considered in total from four different sources for this study. Our core and accessory genomic epidemiology analyses showed that grass isolates cluster in seven groups well differentiated from one another and from the major human and animal isolates. Furthermore, we found new sequence types under both multilocus sequence typing schemes: two under the Pasteur scheme and seven for the Oxford scheme. The grass isolates contained fewer antibiotic-resistance genes and were not resistant to the antibiotics tested. Our results demonstrate that these novel clones appear to have limited antibiotic resistance potential. Given our findings, we propose that genomic epidemiology and surveillance of *A. baumannii* should go beyond the hospital settings and consider the environment in an explicit One Health approach.

## Data Summary

The newly sequenced genomes are listed in Table S2, these genomes have been submitted to the NCBI and their BioProject number is PRJNA905713.

The human and animal publicly available genomes used in this study are listed in Table S3. All the supplementary tables are available in the Supplementary Material File.

**Impact Statement**

This study is very important as it shows that grass can be the habitat (and reservoir) of the important ‘nosocomial pathogen’ *A. baumannii *. Our study is also relevant in identifying the differences between the genomes of the environmental *A. baumannii* and the clinical clones distributed globally. In a broader context, our study highlights the importance of a One Health perspective when tracking the transmission dynamics of *A. baumannii *. These aspects are vital to understanding the epidemiology of *A. baumannii* and potential new sources and origins of outbreaks in the future of climate change and its impact on the nexus between environment and infectious disease. The data and findings will be of interest to clinicians, molecular biologists, those investigating the antimicrobial resistance profiles of *A. baumannii* and researchers investigating the evolution of *A. baumannii* as a nosocomial pathogen.

## Introduction

*A. baumannii* is one of the most clinically relevant hospital-associated pathogens, frequently causing difficult-to-treat multidrug-resistant infections in many clinical settings. The World Health Organization has classified this bacterium as the most important pathogen, for which new antibiotics need to be developed [1]. For many years, *A. baumannii* was regarded as a nosocomial pathogen, which was rarely found in non-clinical settings [2]. The ability of *A. baumannii* but no other species within the genus *Acinetobacter* to exist in non-clinical environments has been described as a misconception [2, 3]. However, in recent years, some studies have clearly demonstrated that these bacteria can be isolated from non-clinical settings [4–9].

For instance, isolates recovered from different animals (birds, cattle, pigs, etc.) have been reported [6, 7, 9]. One study identified *Acinetobacter* species in 17 % of the vegetables analysed with *A. baumannii* identified as the commonest [10]. Another study, using several molecular typing strategies (MLST and the gene *bla*_OXA-51_), found this bacterium in water, milk, meat, cheese and animal samples [7]. A very recent study even found *A. baumannii* in a milk tank [11]. Clearly, isolates of this pathogen have been found in different types of environments [4, 6, 7, 9, 12]. However, to the best of our knowledge, soil and related plant environments have received little attention.

Although isolates from diverse non-clinical settings have been studied, no attempt has been made to analyse if grass can be part of the natural environment for this bacterium and a potential reservoir. Here, employing genome sequencing and antimicrobial susceptibility tests we analyse 61 isolates sampled from a field trial in Ireland collected for 18 weeks in the summer of 2019. To provide a better framework, in our phylogenomic approach we included some recently described novel animal lineages and representative genomes from the major international clones (ICs) circulating in the clinic [6]. Our results show that grass isolates form novel clones not related to human and animal clones and with limited antibiotic resistance potential. From an ecological point of view, our study shows that grass can be a habitat for some populations (clones/lineages) of this species.

## Methods

### Field trial, manure spreading and sampling, and bacterial identification

In the summer of 2019, a field trial was carried out in Johnstown Castle, Co. Wexford, Ireland (52.294117,–6.501027). No animals had been grazed on the field for 7 months prior to the beginning of the trial. In the field, 1 m^2^ plots were established in a randomized complete block design. Each plot had a 1.5 m buffer zone to avoid cross-contamination between treatments. Each plot was designated one of four treatments: untreated (control), pig manure (SM), cow manure (CM) and poultry manure (PM). Manures were collected from three separate farms and stored outside, out of direct sunlight, for 3 weeks before spreading. Four biological replicates of each treatment were analysed across 11 sampling points. Grass in the trial area was trimmed to approximately 5 cm in length before manure spreading, mimicking grazing height. The trimmers were disinfected using 70 % ethanol between each section. From the grass trimmed from the field trial site, three 200 g (fresh weight) biological replicates were taken as background samples (Timepoint BM). Manure was spread on 25 June 2019, at the start of the field trial (Table S1, available in the Supplementary Material File). Following manure spreading, grass samples were collected at ten additional time points (T0-T9) (Table S1). Then, we carried out bacterial isolation and identification of the isolates via MALDI-TOF and 16S rRNA sequencing see File S1 for details.

### Antimicrobial susceptibility testing

*A. baumannii* were tested for resistance to imipenem (10 µg), amikacin (30 µg), ciprofloxacin (5 µg) and ceftazidime (30 µg). Imipenem, amikacin and ciprofloxacin testing were according to EUCAST guidelines for *A. baumannii* [13]. The ceftazidime susceptibilities were interpreted using breakpoints according to CLSI guidelines for *A. baumannii* [14].

### Genome sequencing, genome assembly and species assignation

Genome sequencing of the grassland isolates was conducted at Novogene, Sacramento, CA 95826, USA. The sequencing was carried out using the NovaSeq 6000 and setting a 150 bp pair-end configuration. We used Trim Galore v0.6.4 to remove the adapters and poor-quality bases. The genomes were assembled via SPAdes v3.13.1 and annotated with Prokka v1.12 [15, 16]. We used CheckM v1.1.3 [17] to corroborate completeness and no contamination. We used Quast v2.3 to calculate the assembly statistics. To corroborate that the isolates belong to the species an ANI analysis was conducted against the reference strain ATCC19606 using pyani [18], whereas the PubMLST database was used for ST assignation [19]. The genome assembly statistics, the ANI value against the reference strain, as well as the ST assignation for each grassland isolate are provided in Table S2, available in the Supplementary Material File.

### Global core phylogeny and gene content analysis

We constructed a core genomes phylogeny without recombination as we did before [20]. Briefly, we identified single gene families (SGFs) without recombination, which then were concatenated to construct a phylogeny. The SGFs were determined from a pangenome analysis employing Roary v3.12.0 [21]; whereas the recombination was established using PhiPack [22]. The phylogeny was constructed via RAxML v8.2.10 [23]. To evaluate the clade support, 100 bootstrap replicates were run. For the gene content analysis, a gene content matrix considering all the isolates was obtained from Roary. Then, every value in the matrix was divided by the sum of all the values and a correlation matrix was produced with the cor() function in R.

### *In silico* resistome, IS presence and virulence gene prediction

An *in silico* prediction of all the antibiotic-resistance genes present in the grass isolates was conducted. Using the proteome of all the grass isolates, we conducted a blast P search against the Comprehensive Antibiotic Resistance Database (CARD) [24]. Only the hits that passed the criteria below were kept. An in-house script was employed to implement at least 95 % identity and 90 % coverage. Then the best match in CARD was selected based on the bit score. We also ran AMRFinderPlus [25], using the same identity and coverage criteria, to identify as many ARGs as possible. The results were visualized using a heat map, which was constructed by means of the packages ComplexHeatmap and MetBrewer in R [26]. Of note, although to infer the resistome we used the proteomes, for simplicity's sake, the heat map presents the genes rather than the proteins. We also screen for ISs in these isolates, via TnCentral [27], considering a 80 % identity and 70 % coverage. For selected strains (see Results), annotated genomes were submitted to the Virulence Factor Analyzer on the Virulence Factor Database [28]. All genomes within the database were used for the analysis. Follow-up analysis of hits was performed using the blast web server.

## Results

### The grass isolates are novel clones of *A. baumannii*

**Fig. 1.**
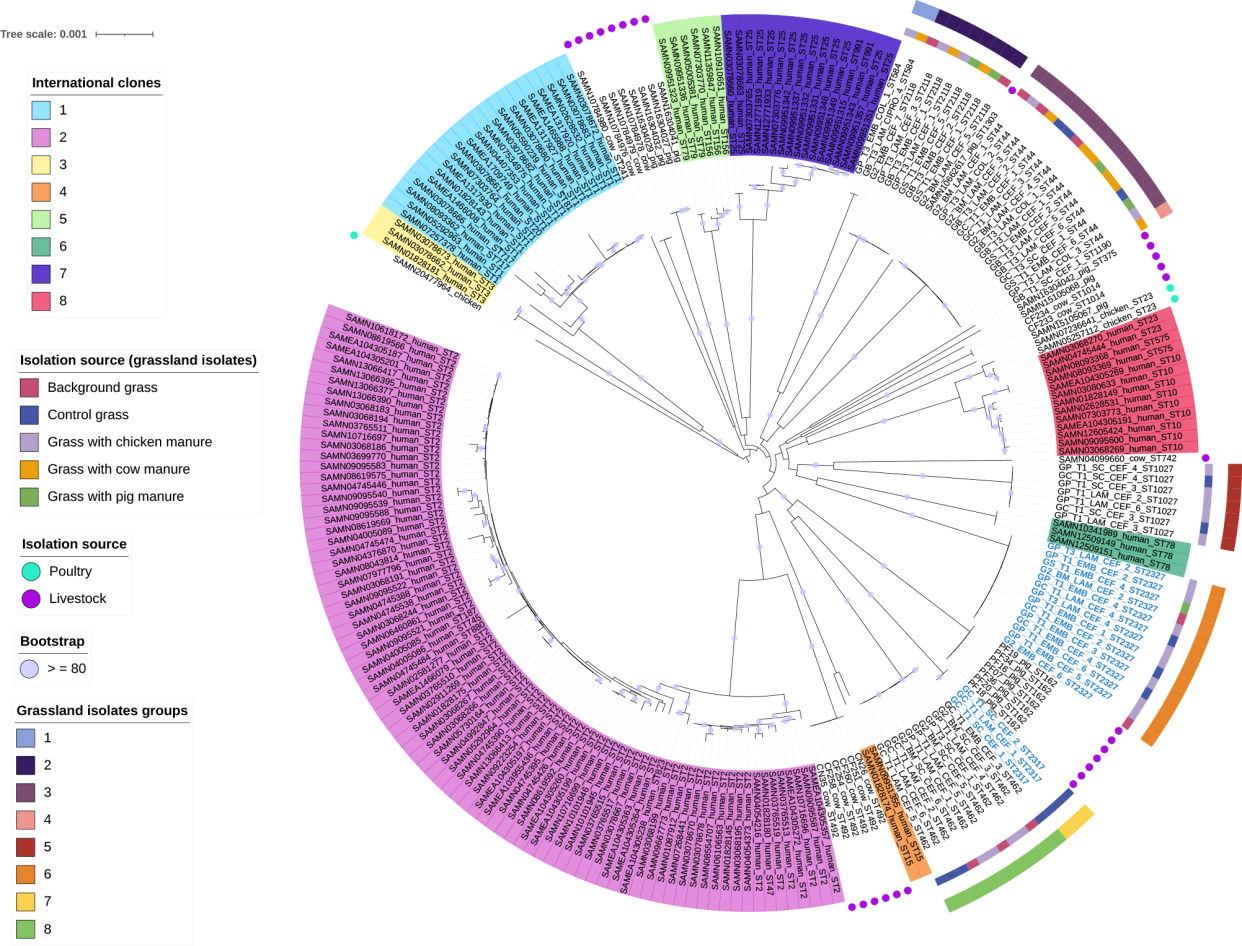
Maximum-likelihood phylogeny based on the single gene families without recombination. The ICs are highlighted in different colours. Animal isolates are marked with dark violet and blue circles. Light blue taxa describe the new STs as per Pasteur scheme. The first ring shows the isolation source, and the most external ring shows the grass groups identified. The violet dots in the internal nodes show bootstrap values≥80 %. The scale bar denotes the substitutions per site.

**Fig. 2.**
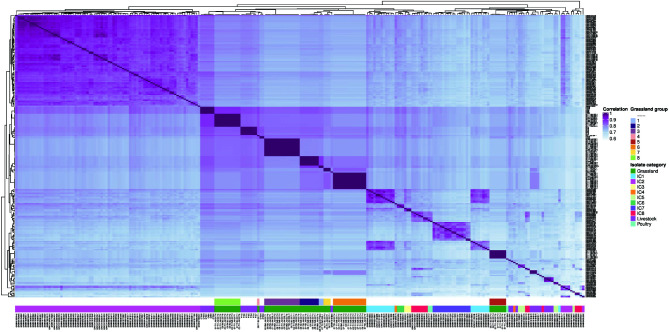
Correlation matrix of gene content. Heat map showing the correlation matrix of the gene content variation among the isolates. The first bottom row depicts the individual grass groups; whereas the second bottom row gives the source (human ICs, animal, grass) for all the isolates.

The isolates were initially identified by means of MALDI-TOF mass spectrometer and 16S rRNA colony PCR and sequencing (see Methods). Nonetheless and as we did previously with clinical isolates [29], we made sure that the isolates are part of *A. baumannii* via an average nucleotide identity analysis (ANI) against the type strain ATCC19606. Table S2 (available in the Supplementary Material File) gives the 61 isolates used for downstream analysis; all of them had ANI values higher than 95%, which is considered the threshold for species assignation. Then, we constructed a core phylogeny to determine the grass isolates' evolutionary relationships (Fig. 1). For this analysis, we included 148 genomes from the main human ICs (see Methods), which represent the main clones circulating in hospitals globally. We also included 31 animal isolates, many of which came from recently described novel lineages of *A. baumannii* [6]. All the extra genomes (human and animal) are provided in Table S3 (available in the Supplementary Material File). In total, 240 genomes were included in the phylogeny. The phylogeny shows that the grass isolates form seven clusters (two or more isolates) and one singleton. All the clusters have strong clade support, as all have bootstrap values equal to or greater than 80. These seven clusters are well differentiated from one another as judged by the long branches that led to each one of them. Remarkably, these seven groups, and the singleton, are not closely related to any of the major ICs. These seven groups are also distantly related to the clusters formed by the animal isolates. Of note, the animal isolates were clustered in a few groups that also are not related to the ICs. Notably, the grass isolates belong to new sequence types never described before (see Table S2). For the Pasteur scheme, these are ST2317 and ST2327; whereas for the Oxford scheme, they are ST2887, ST2888, ST2889, ST2890, ST2891, ST2892 and ST2893. Of note, isolates from cluster 3 were assigned to two Oxford STs due to paralogous genes of gdhB (Table S2). We recently postulated that pan-genomic epidemiology (the use of both core and accessory genomes) could be more powerful than just core genomic epidemiology [30]. Thus, we conducted a gene content analysis considering all the isolates (see Fig. 2 and Methods) to further analyse their evolutionary relationships. Notably, we obtained very similar patterns of clustering as with the core phylogeny. The seven grass groups were recovered with the gene content analysis (Fig. 2, first bottom row). Furthermore, it can also be appreciated that the grass and animal groups are well differentiated from the human clones (Fig. 2, first bottom row). We want to highlight that, even though we sampled a very small geographic area, we found a significant amount of diversity in *A. baumannii* grass clones: seven clusters and one singleton. No *A. baumannii* was isolated from the manure samples in this study. We did not find any clustering according to the type of manure (Fig. 1, internal ring). A total of 25 *A. baumannii* were isolated from the grass with no manure: *A. baumannii* (*n*=10) were isolated from the grass prior to the application of manure (background grass Table S2) and from the control grass (*n*=15) (Table S2). Therefore, grass is the most likely source of *A. baumannii *. Thus, the isolates were most likely of grass origin rather than introduced from the manure. Collectively, these results reveal that several novel clones (lineages) are co-existing in this field trial. Furthermore, these novel lineages are well differentiated from the well-known ICs and even animal isolates. Also, manure does not seem to have an effect on *A. baumannii* presence.

### Antimicrobial resistance profiles and *in silico* prediction of ARGs

**Fig. 3.**
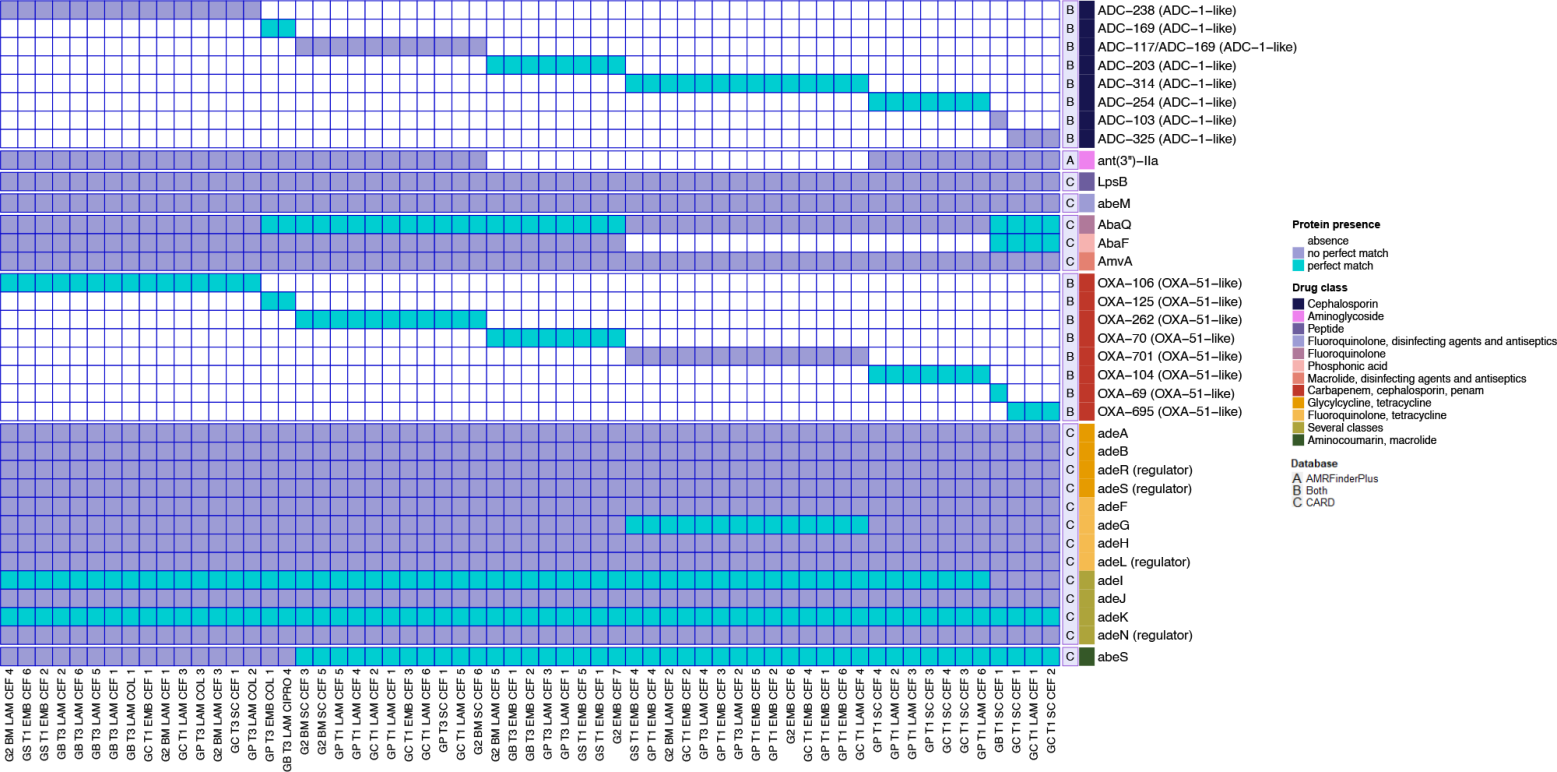
Heat map showing the antibiotic resistance genes per grass isolate. On the right antibiotic-resistance genes with significant matches are shown. Perfect matches are in blue squares, whereas no perfect matches are in violet. Isolate names are provided at the bottom. Drug classes are coloured-coded. For the OXAs and ADCs variants, we put in parentheses the gene families they belong to. It is also highlighted which database identified which ARG: AMRFinderPlus (letter A), CARD (letter C) or identified by both (letter B).

All *A. baumannii* isolated from grass were analysed for resistance to the clinically relevant antimicrobials (ceftazidime, imipenem, ciprofloxacin and amikacin). Two isolates were intermediate to imipenem (GP T1 SC CEF 4 and GB T3 LAM CIPRO 4). Six grass isolates (GC T1 SC CEF 4, GP T1 SC CEF 3, GP T3 LAM CEF 2, GP T1 LAM CEF 3, GP T3 LAM COL 3, GP T1 LAM CEF 2) were intermediate to ceftazidime. However, no isolates were resistant to ceftazidime, imipenem, ciprofloxacin or amikacin. Thus, these grass clones are not resistant to clinically relevant antimicrobials. Then, to identify if the isolates contained any genetic antibiotic resistance determinants, we performed an *in silico* prediction of the resistome of the grassland isolates using the CARD database [24] and also AMRFinderPlus [25] (Fig. 3). Notably, the AMR genes found encode intrinsic resistance, but the specific variant differed across the isolates. While the following genes were identified as AMR genes based on the CARD database they are part of the *A. baumannii* chromosome: the ade family (*adeMHLFGASBRJIKN*) (efflux complexes), *lpsB* (lipopolysaccharide), *AmvA* (efflux), *AbaQ* (efflux) and *abeS* (efflux). These genes or their variants were detected across all isolates. While AbaF is also an efflux component it was absent from 21 isolates. All strains were positive for the intrinsic *ampC* beta-lactamase gene, sometimes referred to as the *Acinetobacter *-derived cephalosporinase (ADC). The ADC genes comprised eight different variants. Eight different oxacillinase (OXA) genes were detected across the isolates. Every isolate contained one OXA gene and all of them belong to the gene family *bla*OXA-51-like (see Fig. 3). As expected due to their intrinsic chromosomal nature, there is an association between the ADC and the OXA variants. For instance, isolate GB T1 SC CEF 1 was the only isolate to contain OXA-69 and the only one to contain a gene very similar to ADC-103. It was a singleton in the phylogeny and the only ST1190 isolate detected. It clustered closest, but still far away from isolates from pig and cow samples. The absence/presence profile of the AMR genes recovers the same seven groups found in our phylogenomic approach. Taken together, these results show that the grass clones had only intrinsic and not acquired resistance genes. Of note, given the relevance of insertion sequences (ISs) for resistant phenotypes in *A. baumannii *, we also looked for ISs in 30 of these isolates from the seven grass groups, via TnCentral [27], but did not find any significant hits.

### *In silico* analysis of virulence-related genes

To determine if the grass isolates differed from clinical isolates in their virulence gene content, one genome from each clade (eight isolates in total) was submitted to the Virulence Gene Analyzer on the Virulence Gene Database [28]. In general, the grass isolates were found to have a similar complement of virulence-related genes to the 13 clinical isolates used in the analysis (Table S4, available in the Supplementary Material File). The most notable difference between the two groups was that the grass isolates had fewer capsule-related genes on average compared to the clinical isolates [31] (*t*-test, *P*=0.0012). Five of the grass isolates also encoded three different type IV pili genes (one in GS T1 EMB CEF 4, one in GP T3 EMB COL 1, one in G2 BM LAM CEF 5 and G2 BM SC CEF 3, and G2 BM LAM CEF 4). While these were not found in the clinical isolates used by the Virulence Gene Analyzer, subsequent blastn analysis of the sequences identified them in 2, 31 and 13 *A *. *baumannii* strains respectively, suggesting they are relatively uncommon across the species. Thus, it seems that in terms of virulence-related genes, the grass isolates are not very different from the human clinical isolates.

## Discussion

Reports of the ubiquity of *A. baumannii* in natural environments, such as soil and water, were considered misconceptions for some time [2, 3]. Likely, these statements arose due to a lack of data on the evolutionary and epidemiological links between the environmental and clinical isolates rather than evidence that this species does not exist in these environments. *A. baumannii* has been mainly studied as a hospital-associated organism since its emergence as a nosocomial pathogen in the 1970s. Nonetheless, over the last two decades, studies have shown that these bacteria can be isolated from non-clinical settings [4–9]. Yet, many of these studies have focused on animal or animal-related products and a lack of effort is evident in plant and soil environments. In this regard, our study focuses on grass, an environment hardly ever explored as a habitat/reservoir of *A. baumannii *. To the best of our knowledge, this is the first study that has sequenced *A. baumannii* isolates from grass. Importantly, compared to other studies related to soil/plant isolates that considered very few isolates (just one or two) from those environments [4, 5, 12], we analyse over 60 isolates from grass.

The diversity of *A. baumannii* within one field from the grass was as diverse as all *A. baumannii* clones globally and the animal isolates. Thus, the variety of *A. baumannii* in the grass environment of one field is as great as the world’s clinical isolates. Why this diversity exists is not yet clear. However, to study the evolution and diversification of *A. baumannii* we must include such isolates in future studies. Our phylogenomic approach identified seven groups (and a singleton) for these grass isolates, which were not closely related to one another. Importantly, neither the grass isolates nor the animal isolates are part of well-known major human ICs, implying that, at least in these cases, the non-clinical populations of *A. baumannii* differ from those previously described in the clinic. We noted that the type of manure treatment (poultry, cow or pig) does not make a difference in terms of the *A. baumannii* clones found in the grass. We did not find any isolates from the pure manure, which is in contrast with two recent studies that found *A. baumannii* in manure samples [17, 32].

*A. baumannii* from clinical settings frequently contain a range of AMR genes (both intrinsic and acquired resistance), including carbapenemases such as *bla*_OXA-23_ or multi-drug resistance genes, including resistance to aminoglycosides, fluoroquinolones and to lesser extents colistin, tigecycline and sulbactam. None of the grass isolates in this study was resistant to ceftazidime, imipenem, ciprofloxacin or amikacin. While every isolate contained one *bla*_OXA-51 -like_ gene, none displayed carbapenem resistance and no isolates contained clinically relevant AMR genes. The lack of AMR (acquired) genes in the grass clones could aid in understanding the factors which have led to the adaptation of *A. baumannii* as an opportunistic pathogen. In contrast, the grass isolates did not differ much from clinical isolates in their virulence gene content; however, phenotypic virulence assays are required to properly compare the virulence potential of the grass isolates.

In conclusion, our work shows that grass can be a reservoir of a great diversity of *A. baumannii,* which is distantly related to the major ICs and some animal clones. These grass clones were susceptible to almost all the antibiotics tested and have fewer antibiotic-resistance genes than the major ICs. Our data also suggest that the environment is a reservoir (habitat) of novel clones that currently do not pose a threat to human health due to the lack of clinically relevant AMR genes, but which may be of great interest in studying the evolution of *A. baumannii* from environmental bacteria to opportunistic AMR pathogen. Our results call attention to the urgent need for more surveillance and genomic epidemiology studies about non-clinical settings under a One Health framework [31], in particular the natural environment.

## Supplementary Data

Supplementary material 1
