# Supplementary material for: Acinetobacter baumannii from grass: novel but non-resistant clones
Source: Microb Genom. 2023 Jul 13;9(7):mgen001054. doi: 10.1099/mgen.0.001054 (PMC10438806; doi:10.1099/mgen.0.001054)
Supplement: Supplementary material 1 [file mgen-9-1054-s001.pdf]

Supplementary File 1

Bacterial isolation and identification of isolates

Grass and manure samples were analysed for the presence of *A. baumannii*. Each grass sample (100 g) was placed into a sterile 500 ml centrifuge bottle containing 250 ml of sterile phosphate buffered saline (PBS) (Gibco). The grass samples were then sonicated for 5 minutes to isolate the grass microbial biofilm. The manure (1 g) was added to nutrient broth and incubated at 37°C for 24 hours. The grass sonicates and manure enrichment samples then underwent tenfold serial dilutions in sterile PBS. For each sample, 100 µl was used to inoculate Leeds Acinetobacter Media agar supplemented with each respective antibiotic at breakpoint concentrations: imipenem (16 mg/L), cefotaxime (4 mg/L), colistin (4 mg/L) and ciprofloxacin (1 mg/L) for the isolation of *Acinetobacter* spp. [13] Agar plates were incubated at 37°C for 24 - 48 hours. Bacterial colony material of pure cultures was transferred by direct smearing in duplicate onto spots of the MALDI-TOF mass spectrometry (MS) target (MTP ground steel, Bruker Daltonics) with toothpicks. To the dried spots, 1 µL matrix solution (10 mg α-cyano-4-hydroxycinnamic acid, Bruker Daltonics) dissolved in 1 mL acetonitrile-water-trifluoroacetic acid (50:47.5:2.5 (vol/vol/vol), Sigma-Aldrich) was added, and this solution was air-dried. Sample spectra were acquired using a microflex LT MALDI-TOF mass spectrometer (Bruker Daltonics) and the flexControl software (ver. 3.4; Bruker Daltonics). Spectra were classified using the Bruker Taxonomy main spectra database (MBT Compass ver. 4.1. with 8468 spectra present, Bruker Daltonics). Bacterial identification was reported to the species level if the score value was above 2.00 or to the genus level if the score was between 1.70 and 1.99. Any isolates that were not successfully identified using MALDI-TOF MS were identified using 16S rRNA colony PCR and sequencing. To prepare bacterial isolates for PCR, a single colony was suspended in 50 µl double-filtered Milli-Q water in a sterile 0.25 ml PCR tube. The colony suspension was then boiled in a Thermocycler (Eppendorf Mastercycler gradient) at 95°C for 10 minutes. A 1465 bp fragment of the 16S rRNA gene was amplified by PCR by targeting nucleotides 27 to 1492 of the 16S rRNA gene. The fragment was amplified using the primer set 27F (5′-AGAGTTTGATCCTGGCTCAG-3′) and 1492R (5′-TACGGYTACCTTGTTACGACTT-3′). Sequences with ≥ 97% identity were accepted at genus level and samples with ≥ 99% identity were accepted at the species level.

Supplementary Table 1: Table displaying sampling time points completed during the field trial.

Timepoint	Timepoint Description	Date Sampled
Background (BM)	Samples taken the day of manure spreading before any manure applied to the field.	25/06/19
T0	Immediately after manure spreading	25/06/19
T1	1 week following manure spreading	02/07/19
T2	2 weeks following manure spreading	10/07/19
T3	3 weeks following manure spreading	16/07/19
T4	4 weeks following manure spreading	22/07/19
T5	5 weeks following manure spreading	29/07/19
T6	6 weeks following manure spreading	06/08/19
T7	10 weeks following manure spreading	02/09/19
T8	14 weeks following manure spreading	02/10/19
T9	18 weeks following manure spreading	30/10/19

Supplementary Table 2

List of all the isolates employed in this study. The isolation date and source are listed. Also, genome assembly statistics and ST assignation are provided for each isolate; blue denotes new STs

Strain	Biosample	Isolation da	Isolation source	N_contigs	N50	L50	%GC	Size	N_genes	Completen	Contamina	ANI vs refe	Oxford_ST	Pasteur_ST
GS T1 EMB CEF 4	SAMN31927120	7/2/19	Grass with Pig Manure	18	728794	2	38.63	3849335	3694	100	0	97.97961	2889	2327
GP T1 LAM CEF 4	SAMN31927121	7/2/19	Grass with Chicken Manure	17	909758	2	38.97	3907143	3646	100	0.41	97.94763	2887	462
GC T1 LAM CEF 6	SAMN31927122	7/2/19	Control Grass (No Manure)	19	909758	2	38.97	3907592	3645	100	0.41	97.94717	2887	462
GP T3 SC CEF 1	SAMN31927123	7/16/19	Grass with Chicken Manure	19	909758	2	38.97	3907592	3645	100	0.41	97.94168	2887	462
GC T1 SC CEF 1	SAMN31927124	7/2/19	Control Grass (No Manure)	22	448651	4	38.6	3970964	3788	100	0	97.94913	2892	2317
G2 BM SC CEF 6	SAMN31927125	6/25/19	Background Grass (no manure on field)	17	909758	2	38.97	3907143	3645	100	0.41	97.94538	2887	462
GP T1 EMB CEF 1	SAMN31927126	7/2/19	Grass with Chicken Manure	17	2156011	1	38.63	3849381	3693	100	0	97.98184	2889	2327
GP T1 EMB CEF 6	SAMN31927127	7/2/19	Grass with Chicken Manure	19	373660	3	38.63	3849021	3694	100	0	97.97971	2889	2327
GC T1 LAM CEF 4	SAMN31927128	7/2/19	Control Grass (No Manure)	16	2156011	1	38.63	3848722	3693	100	0	97.98181	2889	2327
G2 BM LAM CEF 5	SAMN31927129	6/25/19	Background Grass (no manure on field)	29	386749	4	39.02	3821903	3633	100	0	97.91593	2893	2118
G2 BM SC CEF 3	SAMN31927130	6/25/19	Background Grass (no manure on field)	19	909593	2	38.97	3907427	3647	100	0.41	97.9416	2887	462
GP T1 EMB CEF 4	SAMN31927131	7/2/19	Grass with Chicken Manure	17	2156011	1	38.63	3849494	3694	100	0	97.9808	2889	2327
G2 BM LAM CEF 4	SAMN31927132	6/25/19	Background Grass (no manure on field)	22	530972	2	38.78	4040395	3815	100	0	97.92196	2890/2891	44
G2 BM SC CEF 5	SAMN31927133	6/25/19	Background Grass (no manure on field)	17	909758	2	38.97	3907143	3647	100	0.41	97.94201	2887	462
GP T1 SC CEF 4	SAMN31927134	7/2/19	Grass with Chicken Manure	54	250849	6	38.81	4114740	4001	100	0.41	97.91929	2888	1027
G2 BM LAM CEF 2	SAMN31927135	6/25/19	Background Grass (no manure on field)	16	2156011	1	38.63	3849430	3693	100	0	97.98134	2889	2327
GP T1 LAM CEF 2	SAMN31927136	7/2/19	Grass with Chicken Manure	52	250766	6	38.8	4114536	4000	100	0.41	97.92329	2888	1027
GP T1 LAM CEF 5	SAMN31927137	7/2/19	Grass with Chicken Manure	19	909776	2	38.97	3907610	3646	100	0.41	97.94506	2887	462
GP T1 LAM CEF 3	SAMN31927138	7/2/19	Grass with Chicken Manure	53	250849	6	38.81	4114756	4001	100	0.41	97.92289	2888	1027
GC T1 LAM CEF 2	SAMN31927139	7/2/19	Control Grass (No Manure)	19	909758	2	38.97	3907592	3646	100	0.41	97.94156	2887	462
GS T1 EMB CEF 6	SAMN31927140	7/2/19	Grass with Pig Manure	21	530972	2	38.78	4040268	3815	100	0	97.93439	2890/2891	44
GP T1 LAM CEF 1	SAMN31927141	7/2/19	Grass with Chicken Manure	19	909758	2	38.97	3907592	3645	100	0.41	97.9471	2887	462
GS T1 EMB CEF 2	SAMN31927142	7/2/19	Grass with Pig Manure	22	530972	2	38.78	4040795	3816	100	0	97.92168	2890/2891	44
GP T1 SC CEF 3	SAMN31927143	7/2/19	Grass with Chicken Manure	53	250849	6	38.81	4115092	4002	100	0.41	97.92346	2888	1027
GC T1 EMB CEF 3	SAMN31927144	7/2/19	Control Grass (No Manure)	19	909758	2	38.97	3907592	3648	100	0.41	97.94714	2887	462
GC T1 EMB CEF 2	SAMN31927145	7/2/19	Control Grass (No Manure)	17	1947779	1	38.63	3848629	3693	100	0	97.98187	2889	2327
GB T3 LAM CEF 2	SAMN31927146	7/16/19	Grass with Cow Manure	23	530744	2	38.78	4041317	3815	100	0	97.934	2890/2891	44
GB T3 LAM CEF 6	SAMN31927147	7/16/19	Grass with Cow Manure	23	530729	2	38.78	4040922	3815	100	0	97.9341	2890/2891	44
GB T3 EMB CEF 1	SAMN31927148	7/16/19	Grass with Cow Manure	28	386749	4	39.02	3822855	3632	100	0	97.91587	2893	2118
GB T3 LAM CEF 5	SAMN31927149	7/16/19	Grass with Cow Manure	22	530744	2	38.78	4040795	3815	100	0	97.9341	2890/2891	44
GP T3 EMB COL 1	SAMN31927150	7/16/19	Grass with Chicken Manure	43	257008	5	38.98	3851368	3638	100	0	97.86246	1208	584
GB T3 LAM CEF 1	SAMN31927151	7/16/19	Grass with Cow Manure	22	530972	2	38.78	4041303	3816	100	0	97.93335	2890/2891	44
GP T3 LAM CEF 4	SAMN31927152	7/16/19	Grass with Chicken Manure	15	2156011	1	38.63	3849647	3693	100	0	97.97917	2889	2327
GB T3 EMB CEF 2	SAMN31927153	7/16/19	Grass with Cow Manure	26	386749	4	39.02	3821965	3631	100	0	97.91772	2893	2118
GB T3 LAM CIPRO 4	SAMN31927154	7/16/19	Grass with Cow Manure	42	257008	5	38.97	3850304	3638	100	0	97.86288	1208	584
GB T3 LAM COL 1	SAMN31927155	7/16/19	Grass with Cow Manure	20	530729	2	38.78	4040370	3815	100	0	97.93418	2890/2891	44
GP T3 LAM CEF 3	SAMN31927156	7/16/19	Grass with Chicken Manure	27	386749	4	39.02	3822623	3632	100	0	97.91484	2893	2118
GC T1 EMB CEF 1	SAMN31927157	7/2/19	Control Grass (No Manure)	24	530729	2	38.78	4041444	3815	100	0	97.92168	2890/2891	44
GB T1 SC CEF 1	SAMN31927158	7/2/19	Grass with Cow Manure	24	464653	3	38.99	3787902	3574	100	0.14	97.96892	1775	1190
GC T1 SC CEF 4	SAMN31927159	7/2/19	Control Grass (No Manure)	51	250849	6	38.8	4114754	4001	100	0.41	97.92349	2888	1027
GP T1 EMB CEF 3	SAMN31927160	7/2/19	Grass with Chicken Manure	15	2156011	1	38.63	3848803	3693	100	0	97.98179	2889	2327
GP T3 LAM CEF 1	SAMN31927161	7/16/19	Grass with Chicken Manure	26	386749	4	39.02	3822117	3631	100	0	97.91491	2893	2118
GP T3 LAM CEF 2	SAMN31927162	7/16/19	Grass with Chicken Manure	18	728794	2	38.63	3848963	3695	100	0	97.9808	2889	2327
GP T1 EMB CEF 5	SAMN31927163	7/2/19	Grass with Chicken Manure	19	373660	3	38.63	3849021	3694	100	0	97.98182	2889	2327
GC T1 LAM CEF 1	SAMN31927164	7/2/19	Control Grass (No Manure)	22	448651	4	38.6	3971541	3789	100	0	97.94913	2892	2317
GS T1 EMB CEF 5	SAMN31927165	7/2/19	Grass with Pig Manure	28	386749	4	39.02	3822818	3633	100	0	97.91604	2893	2118
GC T1 SC CEF 3	SAMN31927166	7/2/19	Control Grass (No Manure)	52	250766	6	38.8	4113841	4000	100	0.41	97.92308	2888	1027
GC T1 SC CEF 2	SAMN31927167	7/2/19	Control Grass (No Manure)	20	448651	3	38.6	3972463	3788	100	0	97.94882	2892	2317
G2 BM LAM CEF 1	SAMN31927168	6/25/19	Background Grass (no manure on field)	19	530972	2	38.78	4041804	3816	100	0	97.92164	2890/2891	44
GC T1 LAM CEF 3	SAMN31927169	7/2/19	Control Grass (No Manure)	20	530972	2	38.78	4042033	3816	100	0	97.93435	2890/2891	44
GC T1 LAM CEF 5	SAMN31927170	7/2/19	Control Grass (No Manure)	18	909593	2	38.97	3907226	3647	100	0.41	97.94451	2887	462
GP T1 EMB CEF 2	SAMN31927171	7/2/19	Grass with Chicken Manure	17	2156011	1	38.63	3849293	3694	100	0	97.98181	2889	2327
GS T1 EMB CEF 1	SAMN31927172	7/2/19	Grass with Pig Manure	28	386749	4	39.02	3822818	3632	100	0	97.91488	2893	2118
G2 EMB CEF 7	SAMN31927173	6/25/19	Background Grass (no manure on field)	27	386749	4	39.02	3822364	3633	100	0	97.91486	2893	2118
G2 EMB CEF 6	SAMN31927174	6/25/19	Background Grass (no manure on field)	15	2156011	1	38.63	3849511	3693	100	0	97.97922	2889	2327
GP T3 LAM COL 3	SAMN31927175	7/16/19	Grass with Chicken Manure	19	530972	2	38.77	4039195	3815	100	0	97.92182	2890/2891	44
GC T1 EMB CEF 4	SAMN31927176	7/2/19	Control Grass (No Manure)	17	2156011	1	38.63	3849381	3693	100	0	97.98181	2889	2327
G2 BM LAM CEF 3	SAMN31927177	6/25/19	Background Grass (no manure on field)	21	530744	2	38.78	4040268	3814	100	0	97.93439	2890/2891	44
GC T3 SC CEF 1	SAMN31927178	7/16/19	Control Grass (No Manure)	23	530729	2	38.78	4041054	3815	100	0	97.92164	2890/2891	44
GP T3 LAM COL 2	SAMN31927179	7/16/19	Grass with Chicken Manure	22	530744	2	38.78	4040464	3814	100	0	97.92168	2890/2891	44
GP T1 LAM CEF 6	SAMN31927180	7/2/19	Grass with Chicken Manure	52	250766	6	38.81	4115435	4002	100	0.41	97.91923	2888	1027

Supplementary Table 3

Extra genomes were used for the analysis. For all the genomes the BioSample numbers are provided. Regarding the human isolates, their ICs are provided. Animal isolates are highlighted in yellow

Biosample	Iso_source	Category	Host	Strain	Isolation date	Geographic location	Isolation source	IC	Pasteur	Oxford	Completeness	Contaminatio
SAMN07303764	human-related	human-related	Homo sapiens	IHSS3526	2016	Honduras	endobronchial tube	1	1	1590	100	0
SAMN03078680	human-related	human-related	Homo sapiens	AB4991	2008	USA	warwound	1	1	207	100	0
SAMEA1466008	human-related	human-related	Homo sapiens	A388	2002	Greece	missing	1	1	439	100	0
SAMEA1317920	human-related	human-related	Homo sapiens	D81	2010	Australia	wound	1	1	441	100	0
SAMN02628532	human-related	human-related	Homo sapiens	R1B	2011	Saudi Arabia	missing	1	1	441	99.91	0
SAMEA1317927	human-related	human-related	Homo sapiens	RBH3	2002	Australia	endotracheal aspirate	1	1	781	100	0
SAMEA1465988	human-related	human-related	Homo sapiens	6772166	2002	Australia	pus	1	1	781	100	0
SAMN03078672	human-related	human-related	Homo sapiens	AB4448	2007	USA	warwound	1	1	945	100	0.14
SAMN03078683	human-related	human-related	Homo sapiens	AB5674	2009	USA	blood	1	1	945	100	0
SAMEA1317930	human-related	human-related	Homo sapiens	D3208	1997	Australia	blood	1	1	231	100	0
SAMEA1709749	human-related	human-related	Homo sapiens	A297	1984	Netherlands	missing	1	1	231	99.73	0.14
SAMN01828143	human-related	human-related	Homo sapiens	NIPH 290	1994	Czech Republic	urine	1	1	231	100	0
SAMN03078682	human-related	human-related	Homo sapiens	AB5197	2008	USA	severe trauma site	1	1	231	100	0.14
SAMN04407353	human-related	human-related	Homo sapiens	MEX11594	2011	Mexico	pleural fluid	1	1	231	100	0
SAMN05292963	human-related	human-related	Homo sapiens	IHIT25424	2011	Germany	missing	1	1	231	100	1.01
SAMN07257378	human-related	human-related	Homo sapiens	13	2012	Brazil	tracheal aspirate	1	1	231	100	0
SAMN07534975	human-related	human-related	Homo sapiens	237	2016	Togo	cerebrospinal fluid	1	1	231	99.22	1.64
SAMN03078661	human-related	human-related	Homo sapiens	AB3340	2006	USA	blood	1	20	449	100	0
SAMN05590239	human-related	human-related	Homo sapiens	071	2010	Spain	blood	1	81	1868	100	0
SAMN03078666	human-related	human-related	Homo sapiens	AB3927	2007	USA	severe trauma site	1	717	441	100	0
SAMN08093362	human-related	human-related	Homo sapiens	ZQ3	2016	Iraq	blood	1	717	498	100	0.62
SAMN08043814	human-related	human-related	Homo sapiens	ZQ1	2016	Iraq	wound swab	2	2	1052	99.19	0.63
SAMN08619566	human-related	human-related	Homo sapiens	AB1	2012	China	blood	2	2	136	99.59	0.63
SAMN10618172	human-related	human-related	Homo sapiens	O1-3	2018	China	sputum	2	2	136	99.98	0.7
SAMN05292964	human-related	human-related	Homo sapiens	IHIT29106	2014	Germany	missing	2	2	1421	99.98	1.2
SAMEA104305272	human-related	human-related	Homo sapiens	4300STDY7045777	2016	Thailand	missing	2	2	1423	99.85	0.63
SAMN03068194	human-related	human-related	Homo sapiens	2004BJAB10	2004	China	sputum	2	2	1513	99.98	0.62
SAMN08619592	human-related	human-related	Homo sapiens	AB27	2014	China	blood	2	2	1633	99.98	0.63
SAMEA104305260	human-related	human-related	Homo sapiens	4300STDY7045764	2016	Thailand	missing	2	2	1634	99.98	0.78
SAMN10716699	human-related	human-related	Homo sapiens	RCS4	2017	France	throat sample	2	2	1634	99.98	0.63
SAMEA104305201	human-related	human-related	Homo sapiens	4300STDY7045705	2016	Thailand	missing	2	2	1684	99.98	0.63
SAMEA104305197	human-related	human-related	Homo sapiens	4300STDY7045701	2016	Thailand	missing	2	2	1890	99.98	0.63
SAMEA104305199	human-related	human-related	Homo sapiens	4300STDY7045703	2016	Thailand	missing	2	2	1933	99.98	0.63
SAMN04054373	human-related	human-related	Homo sapiens	A186	missing	South Africa	missing	2	2	204	99.59	0.61
SAMN10101945	human-related	human-related	Homo sapiens	14A771	2014	France	respiratory sample	2	2	348	99.59	0.63
SAMN10101946	human-related	human-related	Homo sapiens	15A831	2015	France	respiratory sample	2	2	348	99.59	0.63
SAMEA104305264	human-related	human-related	Homo sapiens	4300STDY7045768	2016	Thailand	missing	2	2	349	99.98	0.77
SAMN01828145	human-related	human-related	Homo sapiens	NIPH 528	1982	Netherlands	urine	2	2	350	99.98	0.61
SAMN03068195	human-related	human-related	Homo sapiens	1999BJAB11	1999	China	sputum	2	2	350	99.92	0.81
SAMN04054216	human-related	human-related	Homo sapiens	Ab Crete	2011	United Kingdom	sputum	2	2	350	99.98	0.61
SAMN06106563	human-related	human-related	Homo sapiens	Ab158_GEIH-2000	2000	Spain	missing	2	2	350	98.63	0.56
SAMN08554707	human-related	human-related	Homo sapiens	6009-2	2010	Portugal	clinical specimen	2	2	350	99.44	0.64
SAMN09095539	human-related	human-related	Homo sapiens	ABAY10007	2010	South Korea	blood	2	2	357	99.56	0.61
SAMN09095540	human-related	human-related	Homo sapiens	ABAY11001	2011	South Korea	blood	2	2	357	99.56	0.71
SAMN03068191	human-related	human-related	Homo sapiens	2011BJAB7	2011	China	sputum	2	2	369	99.98	0.96
SAMN09095583	human-related	human-related	Homo sapiens	ABAY13010	2013	South Korea	blood	2	2	369	99.59	0.61
SAMEA104305187	human-related	human-related	Homo sapiens	4300STDY7045691	2016	Thailand	missing	2	2	436	99.98	0.65
SAMN13066395	human-related	human-related	Homo sapiens	KT_2015_40	2015	Greece	bronchial	2	2	436	99.98	0.65
SAMN03078670	human-related	human-related	Homo sapiens	AB4052	2007	USA	warwound	2	2	452	99.59	1.57

SAMN03078678	human-related	human-related	Homo sapiens	AB4932	2008	USA	sputum	2	2	452	99.59	0.61
SAMN03765515	human-related	human-related	Homo sapiens	MDRAB16	2010	Taiwan	blood	2	2	455	99.32	0.7
SAMN03765513	human-related	human-related	Homo sapiens	KSPU17	2013	Taiwan	sputum	2	2	455	99.59	1.03
SAMEA104305357	human-related	human-related	Homo sapiens	4300STDY7045862	2016	Thailand	missing	2	2	493	99.98	0.63
SAMN04745538	human-related	human-related	Homo sapiens	XH819	2014	China	sputum	2	2	540	99.59	1.57
SAMN08619575	human-related	human-related	Homo sapiens	AB10	2013	China	blood	2	2	540	99.98	0.61
SAMN03765517	human-related	human-related	Homo sapiens	MDRAB41	2009	Taiwan	blood	2	2	544	99.98	0.23
SAMN03765519	human-related	human-related	Homo sapiens	MDRAB58	2009	Taiwan	blood	2	2	545	99.98	0.63
SAMN04745395	human-related	human-related	Homo sapiens	XH515	2014	China	sputum	2	2	547	99.59	0.63
SAMN04745426	human-related	human-related	Homo sapiens	XH665	2015	China	sputum	2	2	547	99.98	0.63
SAMN08619569	human-related	human-related	Homo sapiens	AB4	2015	China	blood	2	2	548	99.98	0.63
SAMN01087912	human-related	human-related	Homo sapiens	NIPH 24	1991	Czech Republic	urine	2	2	556	99.98	0.61
SAMN07268441	human-related	human-related	Homo sapiens	Ab_12	2013	Ivory Coast	urine	2	2	556	98.24	1.13
SAMN03068266	human-related	human-related	Homo sapiens	2011ZJAB1	2011	China	sputum	2	2	643	99.98	0.61
SAMN05730164	human-related	human-related	Homo sapiens	ZJ06-200P5-1	2012	China	missing	2	2	643	99.59	0.67
SAMN03068267	human-related	human-related	Homo sapiens	2011ZJAB2	2011	China	abdominal fluid	2	2	651	99.59	0.61
SAMN03078667	human-related	human-related	Homo sapiens	AB4025	2007	USA	wound	2	2	848	99.98	0.61
SAMN09095588	human-related	human-related	Homo sapiens	ABAY13015	2013	South Korea	blood	2	2	858	99.78	0.61
SAMEA104305363	human-related	human-related	Homo sapiens	4300STDY7045869	2016	Thailand	missing	2	2	938	99.98	0.66
SAMN04745388	human-related	human-related	Homo sapiens	XH508	2014	China	sputum	2	2	191	99.59	0.64
SAMN09095522	human-related	human-related	Homo sapiens	ABAY09003	2009	South Korea	blood	2	2	191	99.98	0.61
SAMN03068186	human-related	human-related	Homo sapiens	2011BJAB3	2011	China	sputum	2	2	218	99.88	1
SAMN03699770	human-related	human-related	Homo sapiens	M3AC14-8	2014	Puerto Rico	skin	2	2	218	99.98	1.27
SAMN03765511	human-related	human-related	Homo sapiens	KMDRAB15	2012	Taiwan	blood	2	2	218	99.59	0.83
SAMN04005089	human-related	human-related	Homo sapiens	AbMDR-GLH5	2011	Spain	tracheobronchial aspirate	2	2	218	100	0
SAMN10716697	human-related	human-related	Homo sapiens	RCS2	2015	France	urine	2	2	218	99.59	0.63
SAMN03068183	human-related	human-related	Homo sapiens	2011BJAB1	2011	China	abdominal fluid	2	2	368	99.98	0.63
SAMN09095521	human-related	human-related	Homo sapiens	ABAY09002	2009	South Korea	blood	2	2	368	99.19	0.67
SAMN03068244	human-related	human-related	Homo sapiens	2011GDAB4	2011	China	blood	2	2	381	99.98	0.61
SAMN06460861	human-related	human-related	Homo sapiens	s228	2012	China	sputum	2	2	381	98.92	0.94
SAMN13066377	human-related	human-related	Homo sapiens	AK_2015_20	2015	Greece	rectal	2	2	425	99.98	0.63
SAMN13066390	human-related	human-related	Homo sapiens	TR_2016_34	2016	Greece	blood	2	2	425	99.88	0.63
SAMN03068199	human-related	human-related	Homo sapiens	2011GDAB1	2011	China	sputum	2	2	457	99.98	0.63
SAMN09667773	human-related	human-related	Homo sapiens	AB4	2011	Hong Kong	missing	2	2	457	99.98	1.04
SAMN04376870	human-related	human-related	Homo sapiens	2015ZJAB3	2014	China	sputum	2	2	784	99.98	0.61
SAMN04745474	human-related	human-related	Homo sapiens	XH722	2013	China	sputum	2	2	784	97.23	0.61
SAMEA1466079	human-related	human-related	Homo sapiens	WM99c	1999	Australia	missing	2	2	208	99.75	0.61
SAMN01828175	human-related	human-related	Homo sapiens	NIPH 2061	2003	Czech Republic	I.V. Cannula	2	2	208	99.98	0.61
SAMN02581277	human-related	human-related	Homo sapiens	BIDMC 56	2013	USA	urine	2	2	208	99.59	0.61
SAMN02911269	human-related	human-related	Homo sapiens	BJ7	2012	China	sputum	2	2	208	99.98	0.63
SAMN03765510	human-related	human-related	Homo sapiens	ISAB51	2010	Taiwan	blood	2	2	208	99.98	0.63
SAMEA103955436	human-related	human-related	Homo sapiens	AC13_M	2015	Morocco	anal margin	2	2	195	99.59	0.7
SAMN04745390	human-related	human-related	Homo sapiens	XH510	2014	China	sputum	2	2	195	99.59	0.63
SAMN04992847	human-related	human-related	Homo sapiens	AB067	2013	India	missing	2	2	195	99.59	0.66
SAMN09223254	human-related	human-related	Homo sapiens	GML-KP7-Col_R_AB	2017	Turkey	endotracheal aspirate	2	2	195	99.98	0.63
SAMN13066413	human-related	human-related	Homo sapiens	GE_2017_62	2017	Greece	missing	2	2	195	99.98	0.63
SAMN01828180	human-related	human-related	Homo sapiens	NIPH 1362	2000	Czech Republic	tracheal aspirate	2	47	437	99.98	0.72
SAMN04005085	human-related	human-related	Homo sapiens	AbMDR-GLH1	2011	Spain	tracheobronchial aspirate	2	187	208	99.92	0.61
SAMN04005086	human-related	human-related	Homo sapiens	AbMDR-GLH2	2011	Spain	tracheobronchial aspirate	2	745	208	99.98	1.44
SAMEA104305238	human-related	human-related	Homo sapiens	4300STDY7045742	2016	Thailand	missing	2	823	457	99.98	0.63
SAMN04745484	human-related	human-related	Homo sapiens	XH733	2013	China	sputum	2	880	208	99.98	0.62
SAMN04745446	human-related	human-related	Homo sapiens	XH685	2014	China	sputum	2	2	451	99.98	0.61

SAMN07977796	human-related	human-related	Homo sapiens	S18	2011	USA	BAL	2	2	451	99.98	0.63
SAMN09095587	human-related	human-related	Homo sapiens	ABAY13014	2013	South Korea	blood	2	2	451	99.98	0.63
SAMN10716696	human-related	human-related	Homo sapiens	RCS1	2014	France	distal bronchial sample	2	2	451	99.98	0.65
SAMN13066417	human-related	human-related	Homo sapiens	SI_2017_69	2017	Greece	broncheal fluid	2	2	451	99.94	0.63
SAMN03078662	human-related	human-related	Homo sapiens	AB3560	2006	USA	blood	3	3	928	100	0
SAMN03078673	human-related	human-related	Homo sapiens	AB4456	2007	USA	trach aspirate	3	3	928	100	0
SAMN01828181	human-related	human-related	Homo sapiens	NIPH 1669	1997	Netherlands	blood	3	3	106	100	0
SAMN09951355	human-related	human-related	Homo sapiens	MC75	2016	Bolivia	ulcer	4	15	236	99.91	0
SAMN01828174	human-related	human-related	Homo sapiens	NIPH 1734	2001	Czech Republic	sputum	4	15	950	100	0
SAMN05005381	human-related	human-related	Homo sapiens	CU032113	2013	USA	wound	5	79	124	100	2.32
SAMN09951323	human-related	human-related	Homo sapiens	MC17	2016	Bolivia	blood	5	79	233	99.95	0.87
SAMN09951336	human-related	human-related	Homo sapiens	MC38	2016	Bolivia	urine	5	79	233	100	0.87
SAMN07303770	human-related	human-related	Homo sapiens	MCR6056	2015	Honduras	blood	5	156	758	100	0.82
SAMN10910651	human-related	human-related	Homo sapiens	A158	2012	Mexico	bronchoalveolar lavage	5	156	758	96.35	2.33
SAMN11359847	human-related	human-related	Homo sapiens	Ab5038	missing	Mexico	missing	5	156	758	99.73	0.82
SAMN10341989	human-related	human-related	Homo sapiens	AB4332	2016	Brazil	respiratory tract	6	78	944	100	0
SAMN12509149	human-related	human-related	Homo sapiens	830	2018	Georgia	missing	6	78	1104	100	0.34
SAMN12509151	human-related	human-related	Homo sapiens	3365	2018	Georgia	missing	6	78	944	100	0.34
SAMN09951331	human-related	human-related	Homo sapiens	MC31	2016	Bolivia	catheter	7	25	1489	100	0
SAMN09951332	human-related	human-related	Homo sapiens	MC32	2016	Bolivia	ulcer	7	25	1489	100	0
SAMN09951348	human-related	human-related	Homo sapiens	MC63	2016	Bolivia	fluid (bone tissue)	7	25	1519	99.86	0
SAMN09951349	human-related	human-related	Homo sapiens	MC64	2016	Bolivia	urine	7	25	1519	99.59	0
SAMN09951351	human-related	human-related	Homo sapiens	MC71	2016	Bolivia	exudate (leg)	7	25	1528	99.73	0
SAMN09951357	human-related	human-related	Homo sapiens	MC77	2016	Bolivia	tracheal secretion	7	25	1528	100	0
SAMN09951337	human-related	human-related	Homo sapiens	MC39	2016	Bolivia	urine	7	25	1529	99.32	0
SAMN09951342	human-related	human-related	Homo sapiens	MC51	2016	Bolivia	lumbosacral ulcer	7	25	1529	100	0
SAMN07303765	human-related	human-related	Homo sapiens	HEU3	2016	Honduras	cornea secretion	7	25	1588	99.91	0
SAMN12771933	human-related	human-related	Homo sapiens	AMA42	2017	missing	missing	7	25	690	100	0.41
SAMN03078660	human-related	human-related	Homo sapiens	AB2828	2006	USA	blood	7	25	993	100	0.55
SAMN03078663	human-related	human-related	Homo sapiens	AB3638	2007	USA	severe trauma site	7	25	993	100	0.41
SAMN07303776	human-related	human-related	Homo sapiens	MCR10179	2015	Honduras	endobronchial tube	7	25	229	100	0
SAMN12771919	human-related	human-related	Homo sapiens	AMA3	2015	missing	missing	7	25	229	100	0.41
SAMN09951315	human-related	human-related	Homo sapiens	MC1	2015	Bolivia	catheter	7	991	1518	100	0.02
SAMN09951343	human-related	human-related	Homo sapiens	MC53	2016	Bolivia	wound	7	991	1518	99	0
SAMEA104305191	human-related	human-related	Homo sapiens	4300STDY7045695	2016	Thailand	missing	8	10	447	100	0.14
SAMN01828149	human-related	human-related	Homo sapiens	NIPH 335	1994	Czech Republic	sputum	8	10	447	100	0.27
SAMN02628531	human-related	human-related	Homo sapiens	LAC-4	1997	USA	missing	8	10	447	100	0.27
SAMN03068269	human-related	human-related	Homo sapiens	2011ZJAB4	2011	China	blood	8	10	447	100	0
SAMN07303773	human-related	human-related	Homo sapiens	MCR9238	2015	Honduras	wound	8	10	447	100	0.21
SAMN09095600	human-related	human-related	Homo sapiens	ABAY14008	2014	South Korea	blood	8	10	447	100	0
SAMN12605424	human-related	human-related	Homo sapiens	TVGH-612	2002	Taiwan	blood	8	10	447	99.73	0.41
SAMEA104305269	human-related	human-related	Homo sapiens	4300STDY7045774	2016	Thailand	missing	8	10	585	100	0.27
SAMN03080633	human-related	human-related	Homo sapiens	T214	2010	Thailand	missing	8	10	585	99.91	0.55
SAMN03068270	human-related	human-related	Homo sapiens	2004ZJAB5	2004	China	sputum	8	23	642	100	0
SAMN04745444	human-related	human-related	Homo sapiens	XH683	2012	China	sputum	8	23	642	99.98	0.68
SAMN08093368	human-related	human-related	Homo sapiens	ZQ9	2016	Iraq	blood	8	575	582	100	0.05
SAMN08093369	human-related	human-related	Homo sapiens	ZQ10	2016	Iraq	cerebrospinal fluid	8	575	582	100	0.05
SAMN16304032	animal origin	livestock	Sus scrofa dom	Res13-Abat-PEA21-	2018	Canada	swab	—	Undetermine	Undetermine	100	0
SAMN10662617	animal origin	livestock	Sus scrofa dom	AB18PR065	2018	China	feces	—	1303	1929	100	0
SAMN16304042	animal origin	livestock	Sus scrofa dom	Res13-Abat-PEA28-	2018	Canada	swab	—	375	Undetermine	100	0
SAMN16304041	animal origin	livestock	Sus scrofa dom	Res13-Abat-PEA25-	2018	Canada	swab	—	Undetermine	Undetermine	100	0
SAMN16304029	animal origin	livestock	Sus scrofa dom	Res13-Abat-PEA16-	2018	Canada	swab	—	Undetermine	Undetermine	100	0

SAMN16304027	animal origin	livestock	Sus scrofa dom	Res13-Abat-EA3-S5-	2018	Canada	swab	---	Undetermined	Undetermine	100	0
SAMN15105067	animal origin	livestock	Sus scrofa	KCJK8630	2018	USA	feces	---	Undetermined	Undetermine	100	0.05
SAMN15105068	animal origin	livestock	Sus scrofa	KCJK8620	2018	USA	feces	---	Undetermined	Undetermine	100	0
SAMN27298904	animal origin	livestock	Sus scrofa dom	PF07	2006/2007	United Kingdom	faecal sample	---	162	235	100	0.07
SAMN27298905	animal origin	livestock	Sus scrofa dom	PF16	2006/2007	United Kingdom	faecal sample	---	162	235	100	0
SAMN27298906	animal origin	livestock	Sus scrofa dom	PF18	2006/2007	United Kingdom	faecal sample	---	162	235	100	0
SAMN27298907	animal origin	livestock	Sus scrofa dom	PF19	2006/2007	United Kingdom	faecal sample	---	162	235	100	0
SAMN27298908	animal origin	livestock	Sus scrofa dom	PF20	2006/2007	United Kingdom	faecal sample	---	162	235	100	0
SAMN27298909	animal origin	livestock	Sus scrofa dom	PF34	2006/2007	United Kingdom	faecal sample	---	162	235	100	0
SAMN27298910	animal origin	livestock	Sus scrofa dom	PF36	2006/2007	United Kingdom	faecal sample	---	162	235	100	0
SAMN10784980	animal origin	livestock	Bos taurus	C3T1-2	2014	Canada	manure	---	241	951	100	0
SAMN10784976	animal origin	livestock	Bos taurus	C1T1-1	2014	Canada	manure	---	Undetermined	Undetermine	100	0.55
SAMN10784979	animal origin	livestock	Bos taurus	C1T1-3	2014	Canada	manure	---	Undetermined	Undetermine	100	0.55
SAMN10784978	animal origin	livestock	Bos taurus	C1T1-2_b	2014	Canada	manure	---	Undetermined	Undetermine	100	0.55
SAMN04099660	animal origin	livestock	Bos taurus	TTU2014-131AME	2014	USA	feces	---	742	1133	100	0
SAMN20477964	animal origin	poultry	Gallus gallus do	LHC22-2	2020	China	feces	---	Undetermined	Undetermine	100	0.48
SAMN27298896	animal origin	livestock	Bos taurus	CF233	2006/2007	United Kingdom	faecal sample	---	1014	Undetermine	100	0.55
SAMN27298897	animal origin	livestock	Bos taurus	CF234	2006/2007	United Kingdom	faecal sample	---	1014	Undetermine	100	0.55
SAMN27298898	animal origin	livestock	Bos taurus	CF251	2006/2007	United Kingdom	faecal sample	---	492	Undetermine	100	0.14
SAMN27298899	animal origin	livestock	Bos taurus	CF254	2006/2007	United Kingdom	faecal sample	---	492	Undetermine	100	0.14
SAMN27298900	animal origin	livestock	Bos taurus	CF258	2006/2007	United Kingdom	faecal sample	---	492	Undetermine	100	0.14
SAMN27298901	animal origin	livestock	Bos taurus	CF260	2006/2007	United Kingdom	faecal sample	---	492	Undetermine	100	0.14
SAMN27298902	animal origin	livestock	Bos taurus	CN26	2006/2007	United Kingdom	nostril sample	---	492	Undetermine	100	0.14
SAMN27298903	animal origin	livestock	Bos taurus	CN35	2006/2007	United Kingdom	nostril sample	---	492	Undetermine	100	0.14
SAMN05257112	animal origin	poultry	Gallus gallus do	65	2012	Germany	missing	---	23	642	100	1.51
SAMN07236641	animal origin	poultry	Gallus gallus do	GB1-2	2015	Germany	missing	---	23	761	100	0.41

Supplementary Table 4

Virulence-related-gene analysis. One genome from each grass group was used for this analysis. Group and ST are stated for these genomes in parentheses.

May_4-5670424684			Predictions																				
**VFclass**	**Virulence factors**	**Related genes**	**GS T1 EMB CEF 4 (Group 6) (ST2327) draft (draft)**	**GC T1 SC CEF 1 (Group 7) (ST2317) draft (draft)**	**G2 BM LAM CEF 5 (Group 2) (ST2118) draft (draft)**	**G2 BM SC CEF 3 (Group 8) (ST462) draft (draft)**	**G2 BM LAM CEF 4 (Group 3) (ST44) draft (draft)**	**GP T1 SC CEF 4 (Group 5) draft (draft)**	**GP T3 EMB COL 1 (Group 1) (ST584) draft (draft)**	**GB T1 SC CEF 1 (Group 4) (ST1190) draft (draft)**	**A.baumannii AB307-0294 chromosome (NC_011595)**	**A.baumannii ACICU chromosome (NC_010611)**	**A.baumannii ATCC 17978 chromosome (NC_009085)**	**A.baumannii AYE chromosome (NC_010410)**	**A.baumannii BJAB07104 chromosome (NC_021726)**	**A.baumannii BJAB0715 chromosome (NC_021733)**	**A.baumannii BJAB0868 chromosome (NC_021729)**	**A.baumannii D1279779 chromosome (NC_020547)**	**A.baumannii MDR-TJ chromosome (NC_017847)**	**A.baumannii MDR-ZJ06 chromosome (NC_017171)**	**A.baumannii SDF chromosome (NC_010400)**	**A.baumannii TCDC-AB0715 chromosome (NC_017387)**	**A.baumannii TYTH-1 chromosome (NC_018706)**
Adherence	Outer membrane protein	ompA	S_1_02840	S_5_02686	S_10_02421	S_11_02877	S_13_02622	S_15_01713	S_32_01862	S_43_02321	ABBFA_000621	ACICU_03089	A1S_2840	ABAYE0640	BJAB07104_03176	BJAB0715_03240	BJAB0868_03135	ABD1_27860	ABTJ_00616	ABZJ_03273	ABSDF0605	ABTW07_3309	M3Q_3327
	Type IV pili biosynthesis(Pseudomonas)	pilA	S_1_02590	-	S_10_01446	S_11_02189	S_13_01986	-	S_32_00866	-	-	-	-	-	-	-	-	-	-	-	-	-	-
Biofilm formation	AdeFGH efflux pump/transport autoinducer	adeF	S_1_01717	S_5_00414	S_10_00321	S_11_01556	S_13_00431	S_15_02887	S_32_01283	S_43_01163	ABBFA_001139	ACICU_02504	A1S_2304	ABAYE1174	BJAB07104_02661	BJAB0715_02706	BJAB0868_02543	ABD1_23030	ABTJ_01214	ABZJ_02696	ABSDF1463	ABTW07_2695	M3Q_2770
		adeG	S_1_01718	S_5_00415	S_10_00322	S_11_01555	S_13_00430	S_15_02886	S_32_01284	S_43_01162	ABBFA_001138	ACICU_02505	A1S_2305	ABAYE1173	BJAB07104_02662	BJAB0715_02707	BJAB0868_02544	ABD1_23040	ABTJ_01213	ABZJ_02697	ABSDF1462	ABTW07_2696	M3Q_2771
		adeH	S_1_01719	S_5_00416	S_10_00323	S_11_01554	S_13_00429	S_15_02885	S_32_01285	S_43_01161	ABBFA_001137	ACICU_02506	A1S_2306	ABAYE1172	BJAB07104_02663	BJAB0715_02708	BJAB0868_02545	ABD1_23050	ABTJ_01212	ABZJ_02698	-	ABTW07_2697	M3Q_2772
	Biofilm-associated protein	bap	-	S_5_00786	-	S_11_01199	S_13_00001;<br>S_13_03806	S_15_03180	S_32_03167	S_43_00789	ABBFA_000777	ACICU_02938	A1S_2696	ABAYE0792	BJAB07104_03029	BJAB0715_03073	BJAB0868_02913	-	ABTJ_00772	-	-	ABTW07_3158	M3Q_3169
	Csu pili	csuA/B	S_1_01629	S_5_00322	S_10_00231	S_11_01657	S_13_00523	-	S_32_01190	S_43_01256	ABBFA_001232	ACICU_02420	A1S_2218	ABAYE1319	BJAB07104_02577	BJAB0715_02616	BJAB0868_02459	ABD1_22150	ABTJ_01303	-	-	ABTW07_2612	M3Q_2687
		csuA	S_1_01628	S_5_00321	S_10_00230	S_11_01658	S_13_00524	-	S_32_01189	S_43_01257	ABBFA_001233	ACICU_02418	A1S_2217	ABAYE1320	BJAB07104_02576	BJAB0715_02615	BJAB0868_02458	ABD1_22140	ABTJ_01304	-	-	ABTW07_2611	M3Q_2686
		csuB	S_1_01627	S_5_00320	S_10_00229	S_11_01659	S_13_00525	-	S_32_01188	S_43_01258	ABBFA_001234	ACICU_02417	A1S_2216	ABAYE1321	BJAB07104_02575	BJAB0715_02614	BJAB0868_02457	ABD1_22130	ABTJ_01305	-	-	ABTW07_2610	M3Q_2685
		csuC	S_1_01626	S_5_00319	S_10_00228	S_11_01660	S_13_00526	-	S_32_01187	S_43_01259	ABBFA_001235	ACICU_02416	A1S_2215	ABAYE1322	BJAB07104_02574	BJAB0715_02613	BJAB0868_02456	ABD1_22120	ABTJ_01306	-	-	ABTW07_2609	M3Q_2684
		csuD	S_1_01625	S_5_00318	S_10_00227	S_11_01661	S_13_00527	-	S_32_01186	S_43_01260	ABBFA_001236	ACICU_02415	A1S_2214	ABAYE1323	BJAB07104_02573	BJAB0715_02612	BJAB0868_02455	ABD1_22110	ABTJ_01307	-	-	ABTW07_2608	M3Q_2683
		csuE	S_1_01624	S_5_00317	S_10_00226	S_11_01662	S_13_00528	-	S_32_01185	S_43_01261	ABBFA_001237	ACICU_02414	A1S_2213	ABAYE1324	BJAB07104_02572	BJAB0715_02611	BJAB0868_02454	ABD1_22100	ABTJ_01308	-	-	ABTW07_2607	M3Q_2682
	PNAG (Polysaccharide poly-N-acetylglucosamine)	pgaA	S_1_01558	S_5_00266	S_10_00161	S_11_01713	S_13_00576	S_15_02350	S_32_01121	S_43_01309	ABBFA_001302	ACICU_02365	A1S_2162	ABAYE1394	BJAB07104_02522	BJAB0715_02547	BJAB0868_02404	ABD1_21610	ABTJ_01379	ABZJ_02516	-	ABTW07_2556	M3Q_2612
		pgaB	S_1_01557	S_5_00265	S_10_00160	S_11_01714	S_13_00577	S_15_02349	S_32_01120	S_43_01310	ABBFA_001303	ACICU_02363	A1S_2161	ABAYE1395	BJAB07104_02521	BJAB0715_02546	BJAB0868_02403	ABD1_21600	ABTJ_01380	ABZJ_02515	-	ABTW07_2555	M3Q_2611
		pgaC	S_1_01556	S_5_00264	S_10_00159	S_11_01715	S_13_00578	S_15_02348	S_32_01119	S_43_01311	ABBFA_001304	ACICU_02362	A1S_2160	ABAYE1396	BJAB07104_02520	BJAB0715_02545	BJAB0868_02402	ABD1_21590	ABTJ_01381	ABZJ_02514	-	ABTW07_2554	M3Q_2610
		pgaD	S_1_01555	S_5_00263	S_10_00158	S_11_01716	S_13_00579	S_15_02347	S_32_01118	S_43_01312	ABBFA_001305	ACICU_02361	-	ABAYE1397	BJAB07104_02519	BJAB0715_02544	BJAB0868_02401	ABD1_21580	ABTJ_01382	ABZJ_02513	-	ABTW07_2553	M3Q_2609
Enzyme	Phospholipase C	plc	S_1_01444;<br>S_1_03568	S_5_00155;<br>S_5_03352	S_10_00043;<br>S_10_03287	S_11_01826;<br>S_11_03223	S_13_00694;<br>S_13_03136	S_15_00506;<br>S_15_02683	S_32_02955;<br>S_32_03264	S_43_02933;<br>S_43_03319	ABBFA_001421;<br>ABBFA_003467	ACICU_00064;<br>ACICU_02247	A1S_0043;<br>A1S_2055	ABAYE1520;<br>ABAYE3825	BJAB07104_00080<br>;<br>BJAB07104_02405	BJAB0715_00082;<br>BJAB0715_02304	BJAB0868_00081;<br>BJAB0868_02287	ABD1_00420;<br>ABD1_20510	ABTJ_01495;<br>ABTJ_03771	ABZJ_00067;<br>ABZJ_02399	ABSDF0054;<br>ABSDF2207	ABTW07_0072;<br>ABTW07_2446	M3Q_2497;<br>M3Q_274
	Phospholipase D	plcD	S_1_03648	S_5_03755	S_10_03598	S_11_03612	S_13_03778	S_15_03834	S_32_03552	S_43_03526	ABBFA_000523	ACICU_03190	A1S_2989	ABAYE0498	BJAB07104_03279	BJAB0715_03342	BJAB0868_03235	ABD1_28800	ABTJ_00508	ABZJ_03374	ABSDF2854	ABTW07_3406	M3Q_3423
Immune evasion	Capsule	Undetermined	S_1_02896;<br>S_1_02898;<br>S_1_03545;<br>S_1_03546;<br>S_1_03547;<br>S_1_03548;<br>S_1_03549;<br>S_1_03550;<br>S_1_03551;<br>S_1_03558;<br>S_1_03559;<br>S_1_03560;<br>S_1_03561	S_5_03359;<br>S_5_03360;<br>S_5_03361;<br>S_5_03362;<br>S_5_03363;<br>S_5_03364;<br>S_5_03366;<br>S_5_03367;<br>S_5_03369;<br>S_5_03373;<br>S_5_03374;<br>S_5_03375;<br>S_5_03376;<br>S_5_03377;<br>S_5_03378;<br>S_5_03379	S_10_03294;<br>S_10_03295;<br>S_10_03296;<br>S_10_03297;<br>S_10_03298;<br>S_10_03299;<br>S_10_03301;<br>S_10_03302;<br>S_10_03307;<br>S_10_03308;<br>S_10_03309;<br>S_10_03310;<br>S_10_03311;<br>S_10_03312;<br>S_10_03313	S_11_03230;<br>S_11_03231;<br>S_11_03232;<br>S_11_03233;<br>S_11_03234;<br>S_11_03235;<br>S_11_03239;<br>S_11_03240;<br>S_11_03241;<br>S_11_03242;<br>S_11_03243;<br>S_11_03244;<br>S_11_03247	S_13_03143;<br>S_13_03144;<br>S_13_03145;<br>S_13_03146;<br>S_13_03147;<br>S_13_03148;<br>S_13_03149;<br>S_13_03150;<br>S_13_03151;<br>S_13_03152;<br>S_13_03154;<br>S_13_03155;<br>S_13_03156;<br>S_13_03157;<br>S_13_03158;<br>S_13_03159;<br>S_13_03160;<br>S_13_03161;<br>S_13_03162	S_15_02657;<br>S_15_02658;<br>S_15_02659;<br>S_15_02660;<br>S_15_02661;<br>S_15_02662;<br>S_15_02663;<br>S_15_02664;<br>S_15_02665;<br>S_15_02666;<br>S_15_02667;<br>S_15_02669;<br>S_15_02670;<br>S_15_02671;<br>S_15_02672;<br>S_15_02673;<br>S_15_02674;<br>S_15_02675;<br>S_15_02676	S_32_02962;<br>S_32_02963;<br>S_32_02964;<br>S_32_02965;<br>S_32_02966;<br>S_32_02967;<br>S_32_02969;<br>S_32_02970;<br>S_32_02975;<br>S_32_02976;<br>S_32_02977;<br>S_32_02978;<br>S_32_02979;<br>S_32_02980;<br>S_32_02981	S_43_02905;<br>S_43_02906;<br>S_43_02907;<br>S_43_02908;<br>S_43_02909;<br>S_43_02910;<br>S_43_02911;<br>S_43_02912;<br>S_43_02913;<br>S_43_02914;<br>S_43_02915;<br>S_43_02916;<br>S_43_02917;<br>S_43_02918;<br>S_43_02921;<br>S_43_02922;<br>S_43_02923;<br>S_43_02924;<br>S_43_02925;<br>S_43_02926	ABBFA_003446;<br>ABBFA_003447;<br>ABBFA_003448;<br>ABBFA_003449;<br>ABBFA_003450;<br>ABBFA_003451;<br>ABBFA_003452;<br>ABBFA_003453;<br>ABBFA_003454;<br>ABBFA_003455;<br>ABBFA_003456;<br>ABBFA_003457;<br>ABBFA_003458;<br>ABBFA_003459;<br>ABBFA_003460;<br>ABBFA_003461	ACICU_00071;<br>ACICU_00072;<br>ACICU_00073;<br>ACICU_00074;<br>ACICU_00075;<br>ACICU_00076;<br>ACICU_00077;<br>ACICU_00078;<br>ACICU_00079;<br>ACICU_00080;<br>ACICU_00081;<br>ACICU_00082;<br>ACICU_00083;<br>ACICU_00084;<br>ACICU_00085;<br>ACICU_00086;<br>ACICU_00087;<br>ACICU_00088;<br>ACICU_00089;<br>ACICU_00090;<br>ACICU_00091;<br>ACICU_00092	A1S_0049;<br>A1S_0050;<br>A1S_0051;<br>A1S_0052;<br>A1S_0053;<br>A1S_0054;<br>A1S_0055;<br>A1S_0056;<br>A1S_0057;<br>A1S_0058;<br>A1S_0059;<br>A1S_0060;<br>A1S_0061;<br>A1S_0062;<br>A1S_0063;<br>A1S_0064;<br>A1S_0065;<br>A1S_0066	ABAYE3800;<br>ABAYE3801;<br>ABAYE3802;<br>ABAYE3803;<br>ABAYE3804;<br>ABAYE3806;<br>ABAYE3807;<br>ABAYE3808;<br>ABAYE3809;<br>ABAYE3810;<br>ABAYE3811;<br>ABAYE3812;<br>ABAYE3813;<br>ABAYE3814;<br>ABAYE3815;<br>ABAYE3816;<br>ABAYE3817;<br>ABAYE3818	BJAB07104_00087<br>;<br>BJAB07104_00088<br>;<br>BJAB07104_00089<br>;<br>BJAB07104_00090<br>;<br>BJAB07104_00091<br>;<br>BJAB07104_00092<br>;<br>BJAB07104_00093<br>;<br>BJAB07104_00094<br>;<br>BJAB07104_00095<br>;<br>BJAB07104_00096<br>;<br>BJAB07104_00097<br>;<br>BJAB07104_00098<br>;<br>BJAB07104_00099	BJAB0715_00090;<br>BJAB0715_00091;<br>BJAB0715_00092;<br>BJAB0715_00093;<br>BJAB0715_00094;<br>BJAB0715_00095;<br>BJAB0715_00096;<br>BJAB0715_00097;<br>BJAB0715_00098;<br>BJAB0715_00099;<br>BJAB0715_00100;<br>BJAB0715_00101;<br>BJAB0715_00102;<br>BJAB0715_00103;<br>BJAB0715_00104;<br>BJAB0715_00105;<br>BJAB0715_00106;<br>BJAB0715_00107;<br>BJAB0715_00108;<br>BJAB0715_00109;<br>BJAB0715_00110;<br>BJAB0715_00111;<br>BJAB0715_00112;<br>BJAB0715_00113;<br>BJAB0715_00114;<br>[illegible]	BJAB0868_00088;<br>BJAB0868_00089;<br>BJAB0868_00090;<br>BJAB0868_00091;<br>BJAB0868_00092;<br>BJAB0868_00093;<br>BJAB0868_00094;<br>BJAB0868_00095;<br>BJAB0868_00096;<br>BJAB0868_00097;<br>BJAB0868_00098;<br>BJAB0868_00099;<br>BJAB0868_00100;<br>BJAB0868_00101;<br>BJAB0868_00102;<br>BJAB0868_00103;<br>BJAB0868_00104;<br>BJAB0868_00105;<br>BJAB0868_00106;<br>BJAB0868_00107;<br>BJAB0868_00108;<br>BJAB0868_00109;<br>BJAB0868_00110	ABD1_00490;<br>ABD1_00500;<br>ABD1_00510;<br>ABD1_00520;<br>ABD1_00530;<br>ABD1_00540;<br>ABD1_00550;<br>ABD1_00560;<br>ABD1_00570;<br>ABD1_00580;<br>ABD1_00590;<br>ABD1_00600;<br>ABD1_00610;<br>ABD1_00620;<br>ABD1_00630;<br>ABD1_00640;<br>ABD1_00650;<br>ABD1_00660;<br>ABD1_00670;<br>ABD1_00680;<br>ABD1_00690	ABTJ_03743;<br>ABTJ_03744;<br>ABTJ_03745;<br>ABTJ_03746;<br>ABTJ_03747;<br>ABTJ_03748;<br>ABTJ_03749;<br>ABTJ_03750;<br>ABTJ_03751;<br>ABTJ_03752;<br>ABTJ_03753;<br>ABTJ_03754;<br>ABTJ_03755;<br>ABTJ_03756;<br>ABTJ_03757;<br>ABTJ_03758;<br>ABTJ_03759;<br>ABTJ_03760;<br>ABTJ_03761;<br>ABTJ_03762;<br>ABTJ_03763;<br>ABTJ_03764	ABZJ_00075;<br>ABZJ_00076;<br>ABZJ_00077;<br>ABZJ_00078;<br>ABZJ_00079;<br>ABZJ_00080;<br>ABZJ_00081;<br>ABZJ_00082;<br>ABZJ_00083;<br>ABZJ_00084;<br>ABZJ_00085;<br>ABZJ_00086;<br>ABZJ_00087;<br>ABZJ_00088;<br>ABZJ_00089;<br>ABZJ_00090;<br>ABZJ_00091;<br>ABZJ_00092;<br>ABZJ_00093;<br>ABZJ_00094;<br>ABZJ_00095;<br>ABZJ_00096;<br>ABZJ_00097	ABSDF0063;<br>ABSDF0064;<br>ABSDF0065;<br>ABSDF0066;<br>ABSDF0067;<br>ABSDF0068;<br>ABSDF0069;<br>ABSDF0070;<br>ABSDF0071;<br>ABSDF0072;<br>ABSDF0073;<br>ABSDF0074;<br>ABSDF0075;<br>ABSDF0076;<br>ABSDF0077;<br>ABSDF0078;<br>ABSDF0079;<br>ABSDF0080;<br>ABSDF0081;<br>ABSDF0082;<br>ABSDF0083	ABTW07_0079;<br>ABTW07_0080;<br>ABTW07_0081;<br>ABTW07_0082;<br>ABTW07_0083;<br>ABTW07_0084;<br>ABTW07_0085;<br>ABTW07_0086;<br>ABTW07_0087;<br>ABTW07_0088;<br>ABTW07_0089;<br>ABTW07_0090;<br>ABTW07_0091;<br>ABTW07_0092;<br>ABTW07_0093;<br>ABTW07_0094;<br>ABTW07_0095;<br>ABTW07_0096;<br>ABTW07_0097;<br>ABTW07_0098;<br>ABTW07_0099;<br>ABTW07_0100	M3Q_281;<br>M3Q_282;<br>M3Q_283;<br>M3Q_284;<br>M3Q_285;<br>M3Q_286;<br>M3Q_287;<br>M3Q_288;<br>M3Q_289;<br>M3Q_290;<br>M3Q_291;<br>M3Q_292;<br>M3Q_293;<br>M3Q_294;<br>M3Q_295;<br>M3Q_296;<br>M3Q_297;<br>M3Q_298;<br>M3Q_299;<br>M3Q_300;<br>M3Q_301;<br>M3Q_302;<br>M3Q_303
	LPS	lpsB	S_1_02267	S_5_02371	S_10_02153	S_11_02620	S_13_02959	S_15_01130	S_32_01577	S_43_02003	ABBFA_003104	ACICU_00440	A1S_0430	ABAYE3345	BJAB07104_00486	BJAB0715_00469	BJAB0868_00489	ABD1_04010	ABTJ_03344	ABZJ_00471	ABSDF3082	ABTW07_0471	M3Q_685
		lpxA	S_1_00069	S_5_00037	S_10_01098	S_11_01888	S_13_00764	S_15_00664	S_32_02142	S_43_02485	ABBFA_001481	ACICU_02088	A1S_1965	ABAYE1587	BJAB07104_01654	BJAB0715_02235	BJAB0868_02224	ABD1_19910	ABTJ_01622	ABZJ_02268	ABSDF1690	ABTW07_2294	M3Q_2434
		lpxB	S_1_00417	S_5_00987	S_10_00762	S_11_00943	S_13_01158	S_15_00323	S_32_00413	S_43_00667	ABBFA_001828	ACICU_01697	A1S_1668	ABAYE1983	BJAB07104_02054	BJAB0715_01882	BJAB0868_01820	ABD1_16550	ABTJ_02010	ABZJ_01859	ABSDF1889	ABTW07_1911	M3Q_2049
		lpxC	S_1_02997	S_5_01459	S_10_01294	S_11_02341	S_13_01766	S_15_02164	S_32_00717	S_43_01705	ABBFA_000151	ACICU_03528	A1S_3330	ABAYE0154	BJAB07104_03624	BJAB0715_03711	BJAB0868_03577	ABD1_32260	ABTJ_00150	ABZJ_03720	ABSDF3466	ABTW07_3736	M3Q_67
		lpxD	S_1_00067	S_5_00039	S_10_01100	S_11_01886	S_13_00762	S_15_00662	S_32_02144	S_43_02483	ABBFA_001479	ACICU_02090	A1S_1967	ABAYE1585	BJAB07104_01652	BJAB0715_02237	BJAB0868_02226	ABD1_19930	ABTJ_01620	ABZJ_02270	ABSDF1688	ABTW07_2296	M3Q_2436
		lpxL	S_1_02266	S_5_02372	S_10_02154	S_11_02621	S_13_02958	S_15_01129	S_32_01578	S_43_02002	ABBFA_003103	ACICU_00441	A1S_0431	ABAYE3343	BJAB07104_00487	BJAB0715_00470	BJAB0868_00490	ABD1_04020	ABTJ_03343	ABZJ_00472	ABSDF3081	ABTW07_0472	M3Q_686
		lpxM	S_1_01979	S_5_00698	S_10_00645	S_11_01290	S_13_00093	S_15_01846	S_32_02597	S_43_00883	-	ACICU_02853	A1S_2609	ABAYE0885	BJAB07104_02942	BJAB0715_02981	BJAB0868_02822	ABD1_25620	ABTJ_00863	ABZJ_03036	ABSDF0888	ABTW07_3075	M3Q_3085
Iron uptake	Acinetobactin	barA	S_1_01790	S_5_00486	S_10_00451	S_11_01484	S_13_00297	S_15_03113	S_32_01404	S_43_01090	ABBFA_001066	ACICU_02574	A1S_2378	ABAYE1100	BJAB07104_02732	BJAB0715_02780	BJAB0868_02614	ABD1_23720	ABTJ_01139	ABZJ_02768	-	ABTW07_2823	M3Q_2841
		barB	S_1_01789	S_5_00485	S_10_00450	S_11_01485	S_13_00298	S_15_03114	S_32_01403	S_43_01091	ABBFA_001067	ACICU_02573	A1S_2375	ABAYE1101	BJAB07104_02731	BJAB0715_02779	BJAB0868_02613	ABD1_23710	ABTJ_01140	ABZJ_02767	-	ABTW07_2822	M3Q_2840
		basA	S_1_01805	S_5_00500	S_10_00465	S_11_01469	S_13_00282	S_15_03098	S_32_01418	S_43_01076	ABBFA_001051	ACICU_02588	A1S_2391	ABAYE1086	BJAB07104_02746	BJAB0715_02794	BJAB0868_02628	ABD1_23850	ABTJ_01124	ABZJ_02782	-	ABTW07_2836	M3Q_2856
		basB	S_1_01804	S_5_00499	S_10_00464	S_11_01470	S_13_00283	S_15_03099	S_32_01417	S_43_01077	ABBFA_001052	ACICU_02587	A1S_2390	ABAYE1087	BJAB07104_02745	BJAB0715_02793	BJAB0868_02627	ABD1_23840	ABTJ_01125	ABZJ_02781	-	ABTW07_2835	M3Q_2855
		basC	S_1_01796	S_5_00492	S_10_00457	S_11_01478	S_13_00291	S_15_03107	S_32_01410	S_43_01084	ABBFA_001060	ACICU_02580	A1S_2384	ABAYE1094	BJAB07104_02738	BJAB0715_02786	BJAB0868_02620	ABD1_23780	ABTJ_01133	ABZJ_02774	-	ABTW07_2829	M3Q_2847
		basD	S_1_01795	S_5_00491	S_10_00456	S_11_01479	S_13_00292	S_15_03108	S_32_01409	S_43_01085	ABBFA_001061	ACICU_02579	A1S_2383	ABAYE1095	BJAB07104_02737	BJAB0715_02785	BJAB0868_02619	ABD1_23770	ABTJ_01134	ABZJ_02773	-	ABTW07_2828	M3Q_2846
		basF	S_1_01793	S_5_00489	S_10_00454	S_11_01481	S_13_00294	S_15_03110	S_32_01407	S_43_01087	ABBFA_001063	ACICU_02577	A1S_2380	ABAYE1097	BJAB07104_02735	BJAB0715_02783	BJAB0868_02617	ABD1_23750	ABTJ_01136	ABZJ_02771	-	ABTW07_2826	M3Q_2844
		basG	S_1_01792	S_5_00488	S_10_00453	S_11_01482	S_13_00295	S_15_03111	S_32_01406	S_43_01088	ABBFA_001064	ACICU_02576	A1S_2379	ABAYE1098	BJAB07104_02734	BJAB0715_02782	BJAB0868_02616	ABD1_23740	ABTJ_01137	ABZJ_02770	-	ABTW07_2825	M3Q_2843
		basH	S_1_01788	S_5_00484	S_10_00449	S_11_01486	S_13_00299	S_15_03115	S_32_01402	S_43_01092	ABBFA_001068	ACICU_02572	A1S_2374	ABAYE1102	BJAB07104_02730	BJAB0715_02778	BJAB0868_02612	ABD1_23700	ABTJ_01141	ABZJ_02766	-	ABTW07_2821	M3Q_2839
		basI	S_1_01787	S_5_00483	S_10_00448	S_11_01487	S_13_00300	S_15_03116	S_32_01401	S_43_01093	ABBFA_001069	ACICU_02571	A1S_2373	ABAYE1103	BJAB07104_02729	BJAB0715_02777	BJAB0868_02611	ABD1_23690	ABTJ_01142	ABZJ_02765	-	ABTW07_2820	M3Q_2838
		basJ	S_1_01786	S_5_00482	S_10_00447	S_11_01488	S_13_00301	S_15_03117	S_32_01400	S_43_01094	ABBFA_001070	ACICU_02570	A1S_2372	ABAYE1104	BJAB07104_02728	BJAB0715_02776	BJAB0868_02610	ABD1_23680	ABTJ_01143	ABZJ_02764	-	ABTW07_2819	M3Q_2837
		bauA	S_1_01797	S_5_00493	S_10_00458	S_11_01477	S_13_00290;<br>S_13_01624	S_15_01563;<br>S_15_03106	S_32_01411	S_43_01083	ABBFA_001059	ACICU_02581	A1S_2385	ABAYE1093	BJAB07104_02739	BJAB0715_02787	BJAB0868_02621	ABD1_23790	ABTJ_01132	ABZJ_02775	-	ABTW07_2830	M3Q_2848
		bauB	S_1_01798	S_5_00494	S_10_00459	S_11_01476	S_13_00289	S_15_03105	S_32_01412	S_43_01082	ABBFA_001058	ACICU_02582	A1S_2386	ABAYE1092	BJAB07104_02740	BJAB0715_02788	BJAB0868_02622	ABD1_23800	ABTJ_01131	ABZJ_02776	-	ABTW07_2831	M3Q_2849
		bauC	S_1_01800	S_5_00496	S_10_00461	S_11_01474	S_13_00287	S_15_03103	S_32_01414	S_43_01080	ABBFA_001056	ACICU_02584	A1S_2388	ABAYE1090	BJAB07104_02742	BJAB0715_02790	BJAB0868_02624	ABD1_23820	ABTJ_01129	ABZJ_02778	-	ABTW07_2833	M3Q_2851
		bauD	S_1_01801	S_5_00497	S_10_00462	S_11_01473	S_13_00286	S_15_03102	S_32_01415	S_43_01079	ABBFA_001055	ACICU_02585	A1S_2389	ABAYE1089	BJAB07104_02743	BJAB0715_02791	BJAB0868_02625	ABD1_23830	ABTJ_01128	ABZJ_02779	-	ABTW07_2834	M3Q_2852
		bauE	S_1_01799	S_5_00495	S_10_00460	S_11_01475	S_13_00288	S_15_03104	S_32_01413	S_43_01081	ABBFA_001057	ACICU_02583	A1S_2387	ABAYE1091	BJAB07104_02741	BJAB0715_02789	BJAB0868_02623	ABD1_23810	ABTJ_01130	ABZJ_02777	-	ABTW07_2832	M3Q_2850
		bauF	S_1_01806	S_5_00501	S_10_00466	S_11_01468	S_13_00281	S_15_03097	S_32_01419	S_43_01075	ABBFA_001050	ACICU_02589	A1S_2392	ABAYE1085	BJAB07104_02747	BJAB0715_02795	BJAB0868_02629	ABD1_23860	ABTJ_01123	ABZJ_02783	-	ABTW07_2837	M3Q_2857
		entE	S_1_01794	S_5_00490	S_10_00455	S_11_01480	S_13_00293	S_15_03109	S_32_01408	S_43_01086	ABBFA_001062	ACICU_02578	A1S_2381	ABAYE1096	BJAB07104_02736	BJAB0715_02784	BJAB0868_02618	ABD1_23760	ABTJ_01135	ABZJ_02772	-	ABTW07_2827	M3Q_2845
	Heme utilization	Undetermined	S_1_01326	S_5_02149	S_10_02624	S_11_00056	S_13_02206	S_15_01352	S_32_02757	S_43_02972	ABBFA_002692	ACICU_00872	A1S_0917	ABAYE2877	BJAB07104_01013	BJAB0715_01024	BJAB0868_01020	ABD1_08690	ABTJ_02892	ABZJ_01011	-	ABTW07_1001	M3Q_1209
		Undetermined	S_1_01325	-	-	-	S_13_01634	-	S_32_02756	S_43_02973	-	ACICU_00873	-	-	-	BJAB0715_01025	BJAB0868_01021	-	-	ABZJ_01012	ABSDF2288	ABTW07_1002	M3Q_1210
		Undetermined	S_1_01324	-	-	-	S_13_01633	-	S_32_02755	S_43_02974	-	ACICU_00874	-	-	-	BJAB0715_01026	BJAB0868_01022	-	-	ABZJ_01013	-	ABTW07_1003	M3Q_1211
		Undetermined	S_1_01323	-	-	-	S_13_01632	-	S_32_02754	S_43_02975	-	ACICU_00875	-	-	-	BJAB0715_01027	BJAB0868_01023	-	-	ABZJ_01014	ABSDF2285	ABTW07_1004	M3Q_1212
		Undetermined	S_1_01322	-	-	-	S_13_01631	-	S_32_02753	S_43_02976	-	ACICU_00876	-	-	-	BJAB0715_01028	BJAB0868_01024	-	-	ABZJ_01015	ABSDF2284	ABTW07_1005	M3Q_1213
		Undetermined	S_1_01321	-	-	-	S_13_01630	-	S_32_02752	S_43_02977	-	ACICU_00877	-	-	-	BJAB0715_01029	BJAB0868_01025	-	-	ABZJ_01016	ABSDF2283	ABTW07_1006	M3Q_1214
		Undetermined	S_1_01320	-	-	-	S_13_01629	-	S_32_02751	S_43_02978	-	ACICU_00878	-	-	-	BJAB0715_01030	BJAB0868_01026	-	-	ABZJ_01017	ABSDF2282	ABTW07_1007	M3Q_1215
		Undetermined	S_1_01318	-	-	-	S_13_01627	-	S_32_02749	S_43_02980	-	ACICU_00880	-	-	-	BJAB0715_01032	BJAB0868_01028	-	-	ABZJ_01019	ABSDF2280	ABTW07_1009	M3Q_1217
		Undetermined	S_1_01317	S_5_02148	S_10_02625	S_11_00057	S_13_02207	S_15_01353	S_32_02748	S_43_02981	ABBFA_002691	ACICU_00881	-	ABAYE2875	BJAB07104_01014	BJAB0715_01033	BJAB0868_01029	ABD1_08700	ABTJ_02891	ABZJ_01020	-	ABTW07_1010	M3Q_1218
		hemO	S_1_01319	-	-	-	S_13_01628	-	S_32_02750	S_43_02979	-	ACICU_00879	-	-	-	BJAB0715_01031	BJAB0868_01027	-	-	ABZJ_01018	ABSDF2281	ABTW07_1008	M3Q_1216
Regulation	Quorum sensing	abaI	S_1_03467	S_5_03556	S_10_03112	S_11_03289	S_13_03205	-	S_32_03018	S_43_02864	ABBFA_003409	ACICU_00136	A1S_0109	ABAYE3761	BJAB07104_00145	BJAB0715_00157	BJAB0868_00152	ABD1_01150	ABTJ_03695	ABZJ_00138	-	ABTW07_0136	M3Q_342
		abaR	S_1_03469	S_5_03554	S_10_03114	S_11_03291	S_13_03207	S_15_02621	S_32_03020	S_43_02862	ABBFA_003407	ACICU_00138	A1S_0111	ABAYE3758	BJAB07104_00147	BJAB0715_00159	BJAB0868_00154	ABD1_01170	ABTJ_03693	ABZJ_00140	-	ABTW07_0138	M3Q_344
	Two-component system	bfmR	S_1_03274	S_5_03140	S_10_02878	S_11_03079	S_13_02829	S_15_02731	S_32_02896	S_43_02686	ABBFA_002866	ACICU_00705	A1S_0748	ABAYE3064	BJAB07104_00755	BJAB0715_00739	BJAB0868_00763	ABD1_07000	ABTJ_03067	ABZJ_00746	ABSDF2719	ABTW07_0736	M3Q_951
		bfmS	S_1_03273	S_5_03139	S_10_02879	S_11_03080	S_13_02828	S_15_02732	S_32_02895	S_43_02687	ABBFA_002865	ACICU_00706	A1S_0749	ABAYE3063	BJAB07104_00756	BJAB0715_00740	BJAB0868_00764	ABD1_07010	ABTJ_03066	ABZJ_00747	ABSDF2718	ABTW07_0737	M3Q_952
Serum resistance	PbpG	pbpG	S_1_03381	S_5_03446	S_10_03213	S_11_03500	S_13_03557	S_15_03413	S_32_03119	S_43_03144	ABBFA_003295	ACICU_00260	A1S_0237	ABAYE3532	BJAB07104_00303	BJAB0715_00285	BJAB0868_00307	ABD1_02210	ABTJ_03537	ABZJ_00287	ABSDF3296	ABTW07_0290	M3Q_503
	LPS rfb locus(Klebsiella)	rmlD	S_1_02897	-	-	-	-	-	-	-	-	-	-	-	-	-	-	-	-	-	-	-	-
Antiphagocytosis	Capsule(Klebsiella)		S_1_02895	-	-	-	-	-	-	-	-	-	-	-	-	-	-	-	-	-	-	-	-
Stress adaptation	Catalase(Neisseria)	katA	-	S_5_01208	-	S_11_03437	-	-	-	-	-	-	-	-	-	-	-	-	-	-	-	-	-
